# Co-designing resources to support the transition from child to adult health services for young people with cerebral palsy: A design thinking approach

**DOI:** 10.3389/fresc.2022.976580

**Published:** 2022-12-16

**Authors:** Jennifer Fortune, Jessica Burke, Conor Dillon, Sally Dillon, Sharon O’Toole, Ann Enright, Annmarie Flynn, Manjula Manikandan, Thilo Kroll, Grace Lavelle, Jennifer M. Ryan

**Affiliations:** ^1^ Department of Public Health and Epidemiology, RCSI University of Medicine and Health Sciences, Dublin, Ireland; ^2^ Advisory Group Contributor, Dublin, Ireland; ^3^ UCD IRIS Centre, School of Nursing, Midwifery and Health Systems, University College Dublin, Dublin, Ireland; ^4^ Institute of Psychiatry, Psychology & Neuroscience, King’s College London, London, United Kingdom

**Keywords:** co-design, design thinking, co-creation, cerebral palsy, transition

## Abstract

**Introduction:**

Design thinking is a human-centred process that aims to identify the needs of end-users and iteratively develop solutions. Involving end-users in the development and design of solutions may enhance effectiveness by increasing focus on the needs of the target population. This paper describes the process of co-designing resources to support the transition from child-centred to adult-orientated health services using a design thinking approach.

**Methods:**

Five co-design workshops were conducted remotely with a young person advisory group and parent advisory group. A design thinking process guided by the Stanford D.School approach was used to understand the transition needs of young people and their parents and iteratively develop solutions to improve end-user experience.

**Results:**

Eight resource prototypes were generated: (1) designated transition coordinator, (2) digital stories of transition experience (3) written informational support (4) transition website, (5) transition checklists and worksheets (6) transition app, (7) transition programme or course and (8) educational programme for health professionals.

**Conclusion:**

Design thinking is a feasible approach to identify, characterise and prioritise resources collaboratively with end-user partners.

## Introduction

Transition from child-centred to adult-orientated health services should be a purposeful, planned process (1) that equips young people with the skills and knowledge to manage their health condition and navigate the adult health care system (2). The phase when young people transfer to an adult health environment coincides with several emotional and social changes, such as developing self-identity, establishing relationships and finding employment (3, 4). Transition may be particularly challenging for young people with cerebral palsy (CP) who report receiving limited information about the transition process, their CP, and about what to expect as they age (5–7). Poor management of the transition process may contribute to poor health outcomes, decreased opportunities for community participation, and decreased quality of life (3, 8, 9). Consequently, there has been increasing interest in developing services, resources and interventions to support young people during this transitional period (10).

Design thinking is a human-centred problem-solving approach to developing solutions to complex problems like transition (11, 12). It prioritizes cultivating empathy and building an understanding of end-user context to effectively integrate their needs and perspectives in the development process (12) thereby improving the likelihood of adoption and sustained use of solutions (13). Through phases of discovery, ideation, prototyping and testing, end-user insights are leveraged to develop best-fit solutions to address their concerns (14).

Design thinking develops empathy with end-users and supports end-user input during various stages of the design process to inform and provide feedback. However, the extent of end-user involvement in the design thinking process can vary for example, from collecting data from informants who contextualise end-user needs, to collecting feedback from end-users who test solutions. Co-design embeds end-users who are experts by experience as equal partners that contribute to the design and decision-making process (15). Co-design promotes active involvement across the whole design process from idea conception to solution refinement (16). A recent review to determine the scope of co-designed healthcare transition interventions among young adults with long-term conditions, including rheumatic, endocrine, and autoimmune conditions, highlighted that active involvement of young people is feasible and may enhance satisfaction with transition care (17). However, to date, there are limited reports describing co-designing transition interventions with young people with CP.

The application of design thinking in health research is in its infancy (11). Specific design processes are poorly described in the existing literature and few studies provide sufficient documentation to allow replication. Increased rigour in reporting design thinking is needed to embed these design practices into research and development (11). This paper describes the application of a design thinking approach to co-design resource prototypes to support the transition from child to adult health services for young people with CP and their families in Ireland.

## Materials and methods

A design thinking approach was informed by findings of the Ignition study; a mixed-methods study to examine the experience of transition among young people with CP (18). Young people with CP aged 16 to 22 years residing in Ireland, in all Gross Motor Function Classification System (GMFCS) levels, their parents and health professionals providing services to people with CP were eligible to participate in the Ignition study. Quantitative and qualitative data were collected through questionnaires and interviews, respectively. Seventy-five young people and/or parents and 108 health professionals completed a questionnaire describing their experience or the provision of key transition practices (19, 20). Twenty-one in-depth semi-structured interviews were conducted with young people and/or parents and twenty-seven interviews were conducted with health professionals.

**Figure 1:**
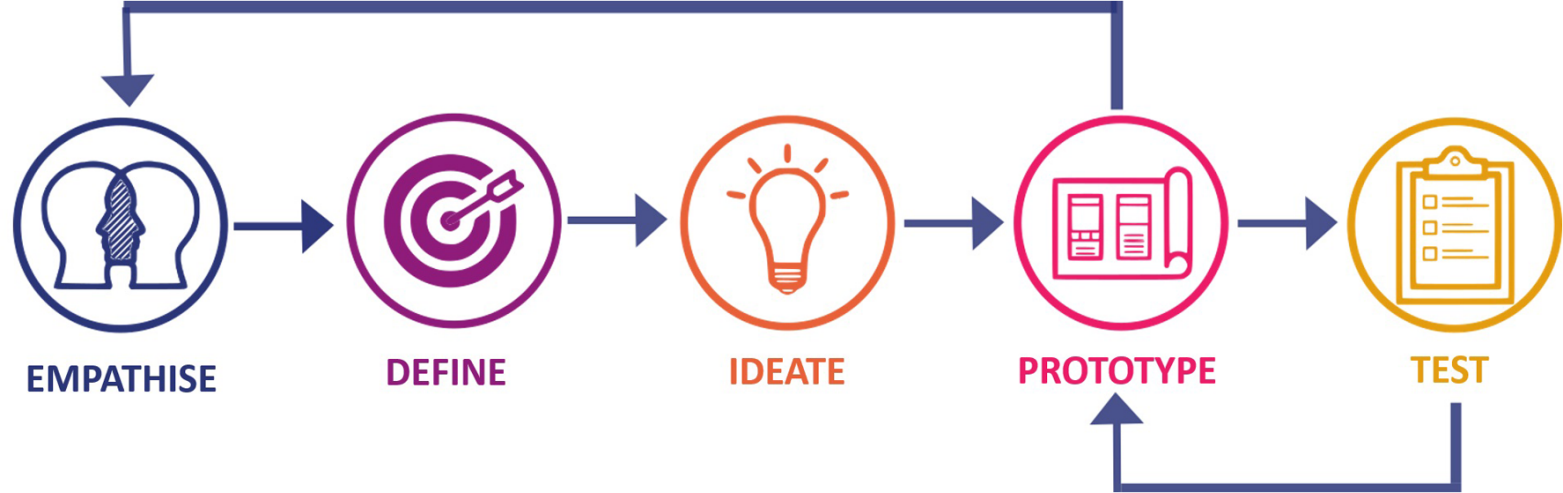
Design thinking stages [adapted from Interaction Design Foundation (21)].

Design thinking uses an iterative approach that includes five stages: Empathise, Define, Ideate, Prototype, and Test (Figure 1) (22). The Empathise stage focuses on connecting with the perspectives and understanding the needs of the end-users. The specific problem(s) to address are determined in the Define stage. During the Ideate stage, a large quantity and wide diversity of ideas are generated to address the problem. Solutions are planned and developed in the Prototype phase. During the Test stage solutions are evaluated for how well they address the problem and refined. We had to make several decisions to operationalise the design thinking process before reaching the Test stage. We will therefore describe our approach, outputs and lessons learned from the Empathise, Define, Ideate and Prototype stages to offer a practical guide to researchers who wish to use a design thinking approach.

### Design team

Our design team consisted of five researchers (JF, JR, MM, TK and GL), a young person's advisory group (YPAG) consisting of four members and a parent's advisory group (PAG) consisting of four members. The researchers are experienced in patient and public involvement (PPI) and co-production. MM, JR, GL and JF have facilitated PPI and advisory groups for children (23) and adults with CP (24). TK is currently leading the Public and Patient Involvement (PPI) Ignite project for University College Dublin (25). GL, JR and JF have co-produced interventions for adults with multiple sclerosis (26) and long term conditions (27). Advisory group members were recruited from Ignition study participants and through snowball sampling. We purposively recruited participants in an attempt to include diversity in relation to geographic area and transition stage.

### Design challenge

Before the process started, the researchers (JF, JR) met to define the *Design Challenge* (28). To establish an open-ended approach to the development of resources we framed the *Design Challenge* as a question:

“How might we improve the experience of transition from child to adult health services for young people with cerebral palsy in Ireland?”

### Co-design workshops

Ten co-design workshops took place in total. Five co-design workshops (28) were conducted with the YPAG and five workshops were conducted separately with the PAG over six months.

Workshops were conducted online due to the COVID-19 pandemic. Technical support, including, written and video guidance and a test call were offered to facilitate online involvement. Where participants were unable to attend, one-to-one catch up sessions were offered. Communications by email or telephone occurred between workshops where required to share workshop notes, obtain further information and validate work completed between sessions by researchers. Workshops were facilitated by at least two researchers. Ground rules for communication and engagement were established to ensure a welcoming and non-judgmental, atmosphere. Workshops were audio-recorded with consent. Recording transcripts, field notes, and photographs of workshop activities were collected. Advisory group members were compensated for their time with vouchers.

Methods and activities to structure each workshop and prime members to think creatively and develop their ideas were chosen from Design Thinking (22, 29) and Human-Centred Design toolkits (28, 30). Toolkit activities undertaken in each workshop are listed in Table 1. Detailed descriptions and the outcomes of each activity are summarized in Supplementary Table S1.

**Table 1:** List of toolkit activities undertaken in co-design workshops.

Workshop	Pre-workshop preparation	1 & 2	3	4 & 5
Design thinking stage		Empathise and Define	Ideate	Prototype
Methods and activities	Frame your design challenge^d^ Build a team^d^	Story share and capture^a^ Saturate and group^b^ Create Insight Statements^d^ Point-of-View (POV)^a^ How Might We Questions^a^^,^^d^	Brainstorm rules^b^^,^^c^ Brainstorming^a^^,^^d^ Bundle ideas^d^ Reality check^c^ Describe idea^c^ Design Principles^d^ 2 × 2 matrix^a^	Describe your concept^a^ Gut check^d^

^a^ Design Thinking Bootleg by the Stanford Design School 2018.

^b^ Bootcamp bootleg by the Stanford Design School.

^c^ Design thinking for Educators by IDEO.

^d^ The Field Guide to Human-Centred Design by IDEO 2015.

### Workshop 1 and 2: Empathise and Define

The purpose of workshop one and two was to build empathy with end-users and gain a greater understanding of their transition needs and priorities. Ignition study survey and interview findings were presented and discussed collaboratively with advisory group members using *Story share and capture* (28). Advisory group members were split into break out rooms and directed to discuss the relevance, importance and meaning of the findings. A researcher joined each breakout room to guide the discussion and record learnings from advisory group members on post-its on Miro board, an online collaborative whiteboard (31). Each post-it contained one concise learning or observation.

Following workshop two, researchers (JF and JR) grouped post-its containing similar learnings together to illuminate patterns and create *Themes* (29). The researchers then summarised each *Theme* to create an *Insight Statement* i.e. a succinct expression of what has been learned from the empathise activities (28). To help clearly articulate the end-user needs, *insight statements* were synthesised by combining three key components user, need, and key learning using a *point of view (POV)* framework (22). Insight statements were reviewed by advisory group members and researchers (GL, MM, TK) over email to ensure they resonated with the *Design Challenge*.

Following collaborative feedback from the advisory group members and researchers, insight statements were used to develop *How Might We (HMW) questions* (22) which are focused questions beginning with the phrase “how might we” used to prompt idea development. Advisory group members were subsequently asked to rate (30) the *HMW questions* in order of importance using an online survey (onlinesurveys.ac.uk).

### Workshop 3: Ideate

**Figure 2:**
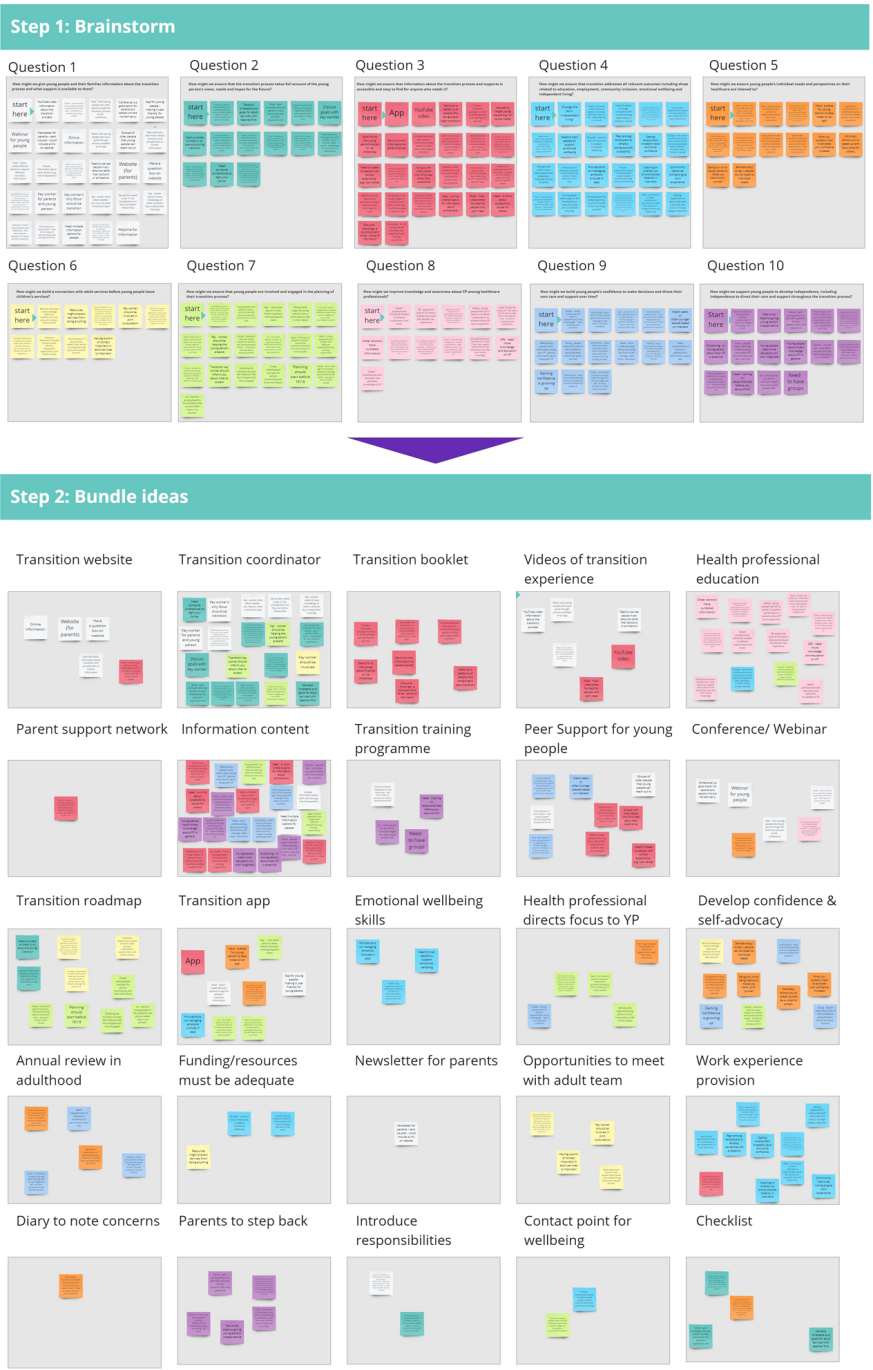
Brainstorm and bundle activities completed in workshop three.

The purpose of workshop three was to *Brainstorm* (28) as many ideas as possible to solve each HMW question. *Ground rules for the brainstorming* session were set by advisory group members (28). Each idea in response to a HMW question was written on a colour-coded post-it on Miro Board (step 1, Figure 2). Post-its containing similar ideas were *bundled* together (28) (step 2, Figure 2). Some ideas, for example “providing information” reoccurred as they addressed multiple HMW questions. The colour coded post-its allowed us to track bundled ideas against the HMW question they were generated in response to.

Following workshop three, a researcher (JF) reviewed notes taken during workshop one and two and added any relevant learnings to the bundled ideas. A *Reality Check* (22) distilled ideas to their core objective and evolved them by merging similar ideas together. Advisory group members evaluated each idea on a scale from one to five based on its achievability and importance through an online survey. Ratings from the YPAG and PAG were combined to determine the ideas that were achievable in the short term, with the potential for high impact. Ratings were plotted on a 2 × 2 matrix, which acted as a decision support tool to determine which ideas to pursue and which to discard (28). Importance, from low to high, was plotted on the y- axis and achievability, from low to high, was plotted on the x-axis. Ideas rated above the median value for importance or achievability were taken forward.

### Workshops 4 and 5: Prototype

The purpose of workshops four and five was to refine ideas and develop resource prototypes. The *Describe your concept* tool (22) elicited feedback from advisory group members. Advisory group members considered the idea's purpose, target user group and *brainstormed* the resource content and format of delivery. Thoughts were recorded on post-its on Miro board. Resource prototypes were examined against the design challenge and the constraints of the project (i.e. time and budget) to determine which prototypes to further iterate and refine.

## Results

Workshops one and four were two-hours duration. All other workshops were three hours duration. The YPAG comprised four young people with CP aged 16–24. The YPAG included three females, and one male, three of whom had already made the transition to adult services. Four female parents formed the PAG of whom three had made the transition to adult health services with their child. Five members attended all workshops, one person missed workshop two, two people missed workshop three, three people missed workshop four, and two people missed workshop five.

### Workshop 1 and 2: Empathise and Define

**Figure 3:**
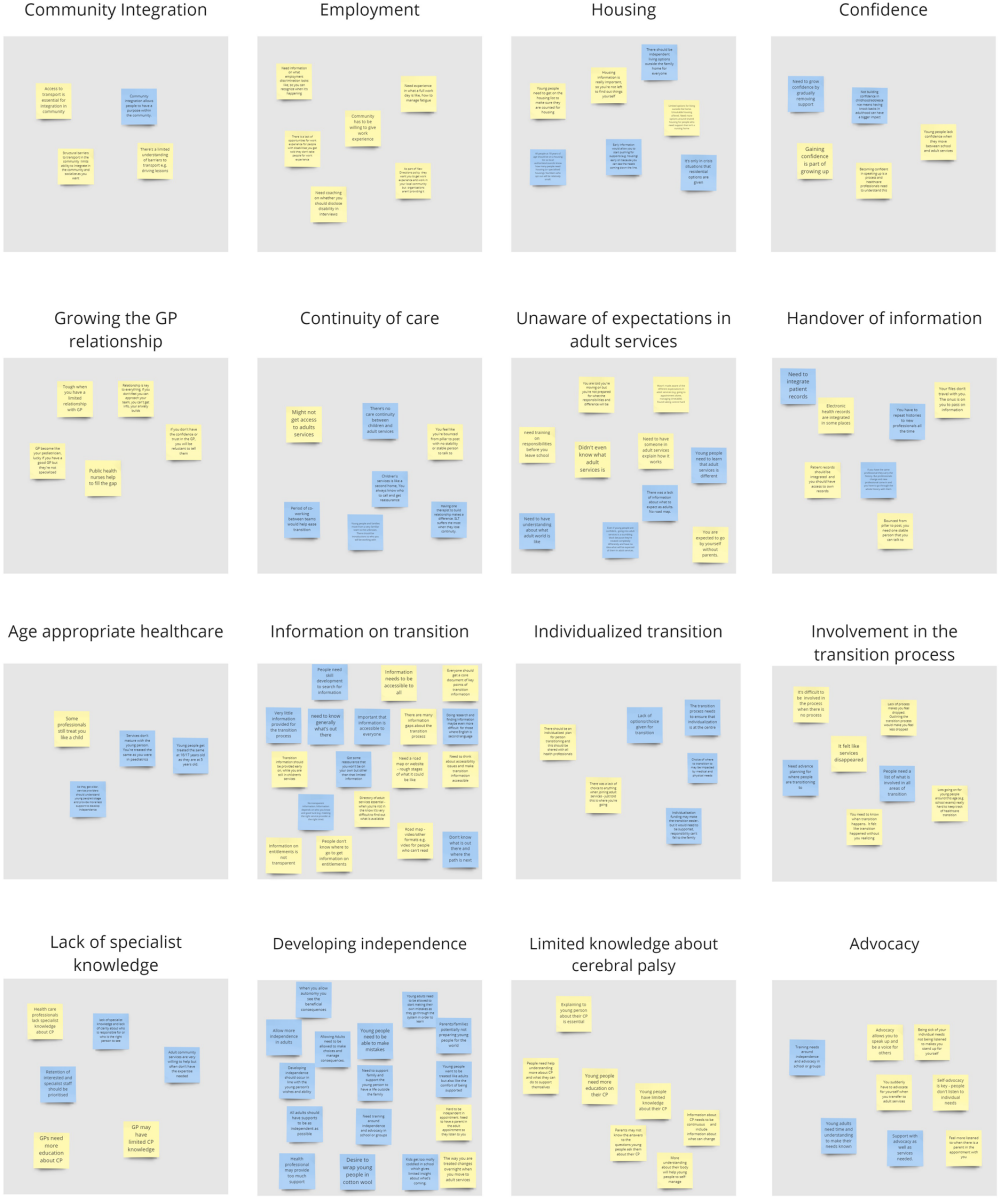
Themes generated by YPAG (yellow post-its) and PAG (blue post-its).

Twenty-five themes were identified. Sixteen themes aligned with the design challenge (Supplementary Table S1; Figure 3). Nine themes did not relate directly to the design challenge and were archived as recommendations (Supplementary Table S3). There was consistency in themes developed from YPAG and PAG learnings, however developing independence and autonomy was more frequently highlighted by the PAG. Challenges to gaining employment, limited relationships with primary care and limited knowledge about their CP were highlighted by the YPAG.

Insight statements and subsequently 19 HMW questions were generated from the 16 themes. Some themes resulted in multiple insight statements and HMW questions (Supplementary Table S1). The perceived importance of each HMW question as rated by the advisory group members is presented in Supplementary Table S1. Given the large number of HMW questions generated, we chose to bring the top ten HMW questions only forward to workshop three.

### Workshop 3: Ideate

During workshop 3, 345 comments were recorded in response to the ten HMW questions (Supplementary Table S4). However, there was overlap in ideas generated across HMW questions. Forty unique ideas were identified (Supplementary Table S1). Although young people and parents brainstormed many similar ideas for resources (*n* = 17), some ideas were identified by the YPAG (*n* = 8) or PAG (*n* = 14) only (Supplementary Table S1). Nineteen ideas that did not address the design challenge (e.g. focused on adult services) or were not possible to achieve within project constraints (e.g. increased funding for transition services) were archived and recorded as health system, adult service or transition service recommendations (Supplementary Table S3). The remaining 20 ideas were evolved by merging similar ideas together to create 13 ideas (Supplementary Table S1). A recurring theme throughout the co-design workshops highlighted the need for accessibility. This formed a *Design Principle* (28) to ensure all resources developed are accessible.

**Figure 4:**
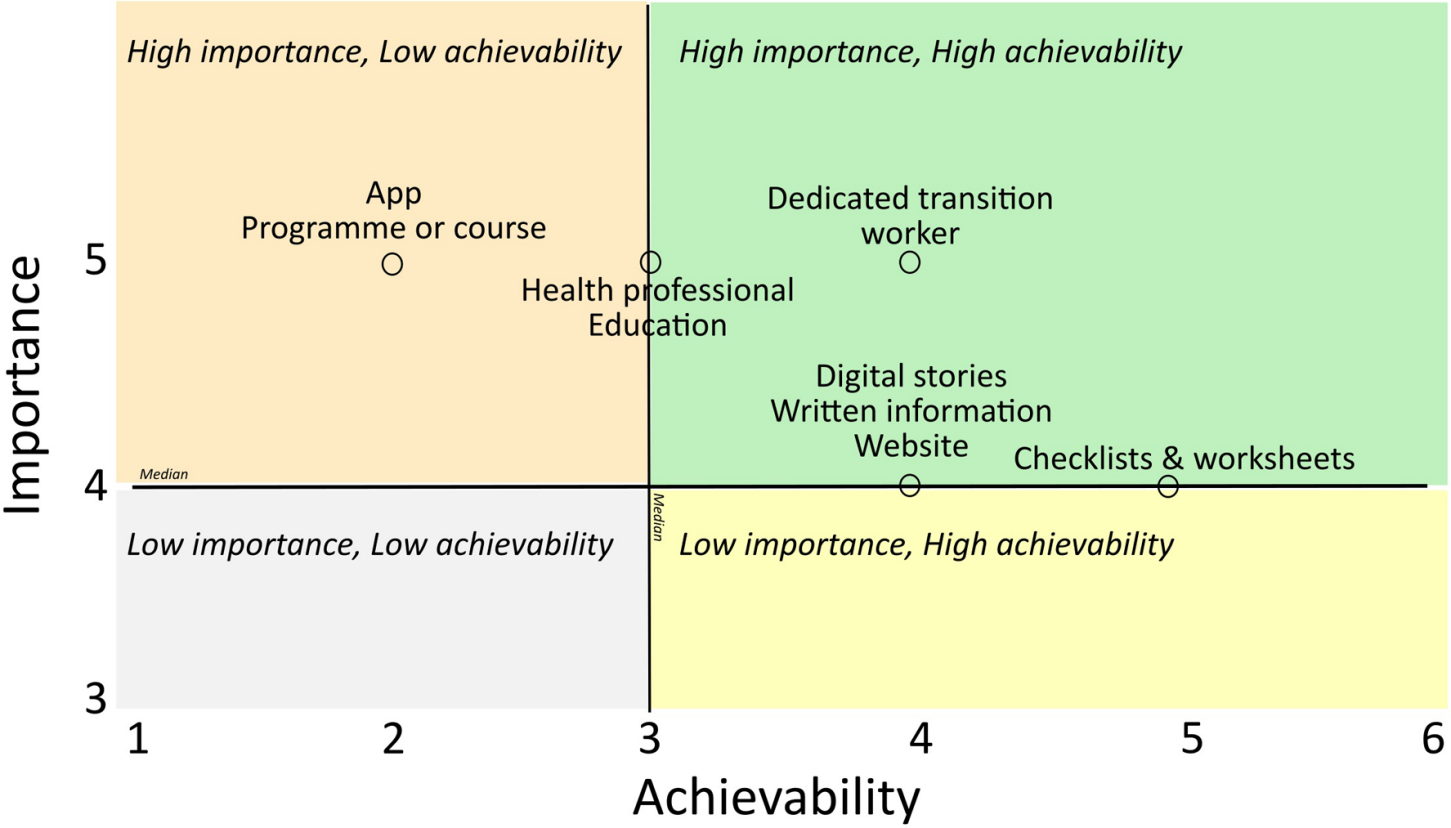
2 × 2 matrix rating achievability and importance of ideas.

How each idea generated by the advisory groups responded to the needs identified was *described* (30) and each idea was summarised in a single sentence (Supplementary Table S1). The perceived importance and achievability of each idea as rated by the YPAG and PAG is presented in a 2 × 2 matrix (Figure 4). The median value for importance was 4. The median value for achievability was 3. The following ideas were rated above the median value for importance or achievability: designated transition coordinator, digital stories of transition experience, written informational support, a transition website, transition checklists and worksheets, a transition App, transition programme or course and educational programme for health professionals.

### Workshops 4 and 5: Prototype

During workshop 4 and 5 each idea's purpose and appropriateness was validated to ensure it legitimately addressed the targeted problem. Advisory group members collaboratively brainstormed to envision the user group, content and format. The research team translated insights gained during these discussions into eight resource prototypes (Supplementary Table S2). These resource prototypes were shared with an advisory group of health professionals with experience of working with young people with CP to obtain feedback and further information that would optimize the utility of the resource in practice. Resource prototypes were assessed by time and budget constraints and narrowed to four for further refinement (1) digital stories of transition experience (2) written informational support (3) a transition website (4) transition checklists and worksheets.

## Discussion

We demonstrated that it is possible to co-design meaningful and useful resource prototypes using a design thinking approach. Actively involving end users in the co-design process provided valuable contextual insights and identified eight resource prototypes to support transition. However during the process we had to make several decisions to ensure the outputs were realistic and manageable.

Design studies often rely on the perceptions of family members or health professionals rather than the perceptions of disabled young people themselves. We chose to hold separate parent and young person workshops. Although common ideas were brainstormed by the YPAG and PAG, including both perspectives offered critical insights of the problem from multiple angles and identified the unique needs and requirements of each group for the transition process. Ideas generated by young people focused on tools to support self-management and autonomy such as transition checklists and transition app. The PAG group highlighted needed changes at the service (e.g. transition planning meeting,) and system level (e.g. transparency of funding allocation). Although holding separate groups involved more time and logistics had we held mixed workshops, these subtle yet important differing needs may not have been identified.

We originally planned to conduct advisory group meetings in person, however the COVID-19 pandemic necessitated the move to online workshops. Remote collaboration reduced travel requirements and facilitated participation of members distributed across Ireland. Not being bound to a physical location allowed members to contribute from their own environment, on their own terms. However, the remote format introduced limitations to interactive, hands-on, co-design methods. We focused on co-design activities that centered on verbal discussion supported by whiteboard materials rather than visualization techniques (e.g. sketching). Introducing alternative mechanisms that target a more diverse range of senses should be considered to engage co-design partners.

We planned to use a series of activities to move sequentially from empathy to prototyping. However, the design thinking process did not follow a linear trajectory. Resource ideas were identified alongside insights in workshop one. Remaining open to discussions that travelled in a non-linear pattern facilitated the creation of ideas that may not have arisen if we remained rigidly within each stage. However, careful documentation of each activity, discussion, idea and decision was essential to enable later recall.

Sifting, refining and discarding ideas that do not meet the design challenge is a recommended part of the design thinking process. Advisory groups defined an extensive list of learnings, insights and ideas that aligned with our scope and aim and consequently narrowing focus proved challenging. To this end we included additional rating activities between sessions to systematically prioritise the most pertinent and feasible ideas. Future teams should consider narrowing focus to a single aspect of the problem and using a criteria-based decision matrix to ensure the most impactful design solutions are selected.

Although the user-focused, design thinking approach offers valuable insights, the process takes time (32). We initially planned to complete the co-design process within six months, however each stage took longer than anticipated. Consideration needs to be given to the commitment and effort required for advisory group members who are contributing their time and expertise over a number of months. Sustaining continued engagement on an ongoing basis through the design process was challenging in the YPAG in light of other personal commitments and circumstances. Flexibility is a key principle to successfully engage people in co-design. Incorporating flexibility to engage in the process at different design stages and reducing focus on same-time interactions by incorporating asynchronicity may overcome participation challenges.

Prototypes of the written information, transition website, transition checklists and digital stories will be iteratively developed with advisory group members in the Test stage to evolve each concept, improve its design and optimise function. Testing sessions will invite feedback from advisory group members on content, design, usability and layout to ensure the developed prototype is reflective of advisory group members' concepts and fits their needs.

### Strengths and limitations

Despite initial concerns, we found it was possible to use a design thinking approach remotely. Breakout rooms, within workshops created a productive environment where members could actively participate in small group discussions before collectively sharing ideas and experiences. Using a virtual whiteboard to present problems, record ideas and summarize discussions facilitated engagement and participation. Facilitation of the workshops by two researchers, allowed one to guide the discussion while the other recorded ideas to ensure no valuable insights were missed.

The number of advisory group members was small. While this approach encouraged active participation and facilitated in-depth contributions, small group numbers may limit the generalisability. Findings may not be representative of other young people with CP or parents whose lived experience and recommendations to improve transition may be different. While we tried to mitigate this limitation by drawing on experiences from the wider Ignition study and employing an iterative approach to develop prototypes relevant to the needs of the end-users, future studies should consider expanding advisory group size where time and facilitation resources allow.

While the design thinking approach created opportunities for collaboration and resulted in the inclusion of perspectives and insights from end users, due to time considerations between-session work was completed by researchers to bundle ideas and refine insights. While the outputs of these activities were shared and validated with advisory group members allowing more time between sessions for feedback and developing opportunities for collaborative synthesis of insights and ideas should be facilitated where possible. Finally, a focus on tangible physical resource prototypes as outputs may have limited exploration of other concepts and solutions.

## Conclusion

In conclusion, this paper presents a detailed description of our co-design process and demonstrates that design thinking can be successfully applied to generate insights, explore new solutions and develop resource prototypes with young people with CP and their families. Researchers should consider design thinking as an approach to engage and gain insight from end-users. We hope that the documentation of our co-creation process, methods and learnings may aid other researchers embarking on their own co-design process.

## Data Availability

The original contributions presented in the study are included in the article/**Supplementary Material**, further inquiries can be directed to the corresponding author/s.
