# Supplementary material for: Co-designing resources to support the transition from child to adult health services for young people with cerebral palsy: A design thinking approach
Source: Front Rehabil Sci. 2022 Dec 16;3:976580. doi: 10.3389/fresc.2022.976580 (PMC9800984; doi:10.3389/fresc.2022.976580)
Supplement: Supplementary file 1 [file Table1.pdf]

Table 1 Description of methods

Workshop	Design thinking stage	Method Name and reference	Description of method	How this method was applied in our study	Learnings & Outcomes
Pre-workshops	Empathise	Frame Your Design Challenge (IDEO.org, 2015, p. 31)	Getting the right frame on your design challenge will get you off on the right foot, organize how you think about your solution and at moments of ambiguity, help clarify where you should push your design. Framing your design challenge is more art than science, but there are a few key things to keep in mind. First, ask yourself: Does my challenge drive toward ultimate impact, allow for a variety of solutions, and take into account context? Dial those in, and then refine it until it's the challenge you're excited to tackle.<br><br>Steps<br>1. Start by taking a first stab at writing your design challenge. It should be short and easy to remember, a single sentence that conveys what you want to do. We often phrase these as questions which set you and your team up to be solution-oriented and to generate lots of ideas along the way.<br><br>2. Properly framed design challenges drive toward ultimate impact, allow for a variety of solutions, and take into account constraints and context. Now try articulating it again with those factors in mind.<br><br>3. Another common pitfall when scoping a design challenge is going either too narrow or too broad. A narrowly scoped challenge won't offer enough room to explore creative solutions. And a broadly scoped challenge won't give you any idea where to start.<br><br>4 Now that you've run your challenge through these filters, do it again. It may seem repetitive, but the right question is key to arriving at a good solution. A quick test we often run on a design challenge is to see if we can come up with five possible solutions in just a few minutes. If so, you're likely on the right track.	A pre-defined objective of the Ignition study was to 'co-produce resources to improve the experience of transition in Ireland.'. JF and JR collaboratively completed the frame your design challenge worksheet(IDEO.org, 2015, p. 33) to develop the design challenge.	We identified the problem we are trying to solve as "improve the experience of transition to adult services in Ireland". We reframed this as a design question, "How might we improve the experience of transition from child to adult health services for young people with cerebral palsy in Ireland". We stated the impact as "develop resources to help young people transition to adult services. Potential constraints identified included time and budget.

Preparation	Empathise	Build A Team (IDEO.org, 2015, p. 35)	Human-centred design works best with cross-disciplinary teams. You could put three business designers to work on a new social enterprise, but if you throw a graphic designer, a journalist, or an industrial designer into the mix, you're going to bring new modes of thinking to your team. It's smart to have a hunch about what kind of talent your team will need—if you're designing a social enterprise, a business designer is probably a good bet—but you won't get unexpected solutions with an expected team.<br><br>Steps:<br>1. First, assess how many team members you'll need, your staff's availability, and when your project should start and end.<br><br>2. Look at the core members of your team and determine what they're good at and what they're not so good at.<br><br>3. Is there a clear technical capability that you'll need but don't currently have—maybe a mechanical engineer, a graphic designer, a skilled writer? Remember that you can always add a team member for<br>a shorter period of time when their skills are most important.	We determined that a minimum of two research staff were required to facilitate each co-design workshop. One researcher facilitated discussion while the other acted as a scribe. We aimed to recruit between 4-6 parents to form a parent advisory group (PAG) and 4-6 young people to form a young person's advisory group (YPAG). We felt this number would allow for communication between members but not be so large as to hamper people's attempts to contribute. We endeavoured to have a diverse group of individuals from different geographical locations in Ireland, of different ages and stages of transition to ensure fair representation of the views and experiences of young people and parents.	Building a team with different experiences, including individuals with lived experience of the transition process enabled examination of the problem from multiple angles and perspectives.

Workshops 1 & 2	Empathise	Story Share and-Capture (d.school, 2018, p. 6)	After interviewing people, bring your team together to share stories that you heard. A story share serves a few purposes. First, it allows team members to get up to speed on what others gathered in the field. Even if everyone was present for the fieldwork, comparing how each person experienced it is valuable. Second, in listening and probing for more information, team members tend to draw out nuance and meaning that wasn't initially realized. This starts the synthesis process.<br>How to story share-and-capture:<br>Unpack observations and share stories that stick out from your team's empathy fieldwork. While each team member shares notes and user stories, others should headline quotes, surprises, and interesting titbits—one headline per post-it.<br>The post-its can be physically grouped and re-grouped on the board to illuminate themes and patterns.<br>The end goal is to understand what's really going on with each user to discover who your users are and what they need in regard to your design space.	Findings from the Ignition study survey and interviews were shared with and discussed by advisory group members to elucidate their impressions, commonalities, differences and concerns. Learnings were captured on post-its on Miro board. Post-its were colour-coordinated. Blue post-its reflected learnings from the PAG. Yellow post-its reflected learnings from the YPAG.	Discussing Ignition study results with the PAG and YPAG illuminated findings and provided a depth of insight that would not have been possible if all team members hadbeen research staff.

Workshop 1 & 2	Define	Saturate and Group (d.school, 2009, p. 14)	HOW to saturate and group<br>You space saturate to help you unpack thoughts and experiences into tangible and visual pieces of information that you surround yourself with to inform and inspire the design team. You group these<br>findings to explore what themes and patterns emerge, and strive to move toward identifying meaningful needs of people and insights that will inform your design solutions.<br>Saturate your wall space (or work boards) with post-its headlining interesting findings (see "Story Share-and-<br>Capture") plus pictures from the field of users you met and relevant products and situations.<br>In order to begin to synthesize the information, organize the post-its and pictures into groups of related<br>parts. You likely have some ideas of the patterns within the data from the unpacking you did when producing the notes. For example, you may have seen and heard many things related to feeling safe, and many things regarding desire for efficiency. Within the group of 'safety', go beyond the theme and try to see if there is a deeper connection that may lead to an insight such as "Feeling safe is more about who I am with than where I am". Maybe there is a relation between groups that you realize as you place items in groups – that safety is often at odds with users' desire for efficiency. Try one set of grouping, discuss (and write down) the findings, and then create a new set of groups. The end goal is to synthesize data into interesting findings and create insights which will be useful to you in creating design solutions.<br>It is common to do the grouping with post-its headlining interesting stories from fieldwork. But grouping is also useful to think about similarities among a group of products, objects, or users.	Post-its created by the YPAG and PAG during "Story Share and Capture were duplicated. Post-its containing similar information were grouped together by JF and JR to form a category. Categories of clustered post-its were given a heading to represent a theme. Themes were shared with and reviewed by advisory group members and researchers (GL, MM, TK) over email.	The following themes emerged:<br>1. Lack of specialist knowledge<br>2. Young people have limited knowledge about CP<br>3. Involvement in the transition process<br>4. Individualised transition<br>5. Information on transition<br>6. Age-appropriate healthcare<br>7. Developing independence<br>8. Confidence<br>9. Advocacy<br>10. Unaware of expectations in adult services<br>11. Continuity of care<br>12. Handover of information<br>13. Growing the GP relationship<br>14. Housing<br>15. Employment<br>16. Community integration<br><br>Themes that did not relate directly to the design brief were archived as recommendations (Supplementary Table 3).
Workshop 1 & 2	Define	Create Insight Statements (IDEO.org, 2015, p. 80)	You've heard a lot from a lot of different people, downloaded learnings, and identified key themes from your research. The next step in the synthesis process is to Create Insight Statements, succinct sentences that will point the way forward. Insight	Themes were used to construct insight statements. To help clearly articulate the end-user needs insights were	The following insight statements were discussed:<br>**Theme**: Lack of specialised knowledge<br>**Insight Statement(s):** :

			statements are incredibly valuable, as they'll help you frame How Might We questions and give shape and form to subsequent Brainstorms It's not always easy to create them, and it will probably take some work editing them down to the three to five main insights that will help you drive toward solutions.<br><br>STEPS<br>01 Take the themes that you identified in Find Themes (p. 80) and put them up on a wall or board.<br>02 Now, take one of the themes and rephrase it as a short statement. You're not looking for a solution here, merely transforming a theme into what feels like a core insight of your research. This is a building block, not a resolved question.<br>03 Once you've done this for all the themes, look back at your original design challenge. Sift through your insight statements and discard the ones that don't directly relate to your challenge. You only want three to five insights statements.<br>04 Take another pass at refining your insights. Make sure that they convey the sense of a new perspective or possibility. Consider inviting someone who is not part of your team to read your insight statements and see how they resonate.	articulated using a point of view (POV framework) which combines the user + need + interesting learning. These insight statements were shared with and reviewed by advisory group members and researchers (GL, MM, TK) over email.	• Young people want specialist knowledge and advice specific to CP however, many adult healthcare professionals are not specialists in physical disability and lack expertise in CP<br><br>**Theme**: Young people have limited knowledge about CP<br>**Insight Statement(s):**<br>• Young people lack insight and awareness about their CP diagnosis and ageing with CP which affects self-management<br><br>**Theme:** Involvement in the transition process<br>**Insight Statement(s):**<br>• Young people feel they are not engaged in planning their transition and that the process happens without their involvement<br><br>**Theme**: Individualised transition<br>**Insight Statement(s):**<br>• Transition often lacks individualisation due to limited choice and not placing the young person at the centre of the process<br><br>**Theme:** Information on transition<br>**Insight Statement(s):**<br>• Young people and parents lack knowledge of the transition process and available support and services<br>• Information about the transition process is often not accessible or transparent
		Point-of-View (POV) (d.school, 2018, p. 21)	A Point of View framework helps outline your design challenge into an actionable problem statement so that you can begin brainstorming solutions. Most importantly, your POV anchors your design thinking project and helps you to articulate your challenge meaningfully<br>How to write a Point of View (POV) statement:<br>After you've interpreted your user empathy interviews, try different ways of stating the problem.<br>POV framework.<br>Start by describing your user in colorful language, including pertinent details. Then, choose your favorite surprise/insight that represents the most powerful shift in your own perspective. Last, articulate what would be game changing for your user, assuming your insight is correct.		**Theme**: Age-appropriate healthcare<br>**Insight Statement(s):**<br>• Young people often feel treated like a child because services do not mature with them<br><br>**Theme:** Developing independence<br>**Insight Statement(s):**<br>• Young people feel unprepared for the sudden change in independence when they move to adult services from the supportive paediatric environment<br><br>**Theme**: Confidence<br>**Insight Statement(s):**

			POV musts.<br>· Make sure your POV flows sensically (so a stranger could comprehend it).<br>· Specifies an insight that's focused on a specific user (rather than a demographic)<br>· Articulates a game-changing direction, without dictating a particular solution.		• Young people lack confidence when they move from child to adult health services<br>• Developing confidence is a process. Health professionals need to understand it takes time<br><br>**Theme:** Advocacy<br>**Insight Statement(s):**<br>• Young people need support to advocate for themselves because they feel their individual needs are not listened to<br><br>**Theme:** Unaware of expectations in adult services<br>**Insight Statement(s):**<br>• Young people and parents are not prepared for the changes to their role and responsibilities in adult services<br>• Young people don't know what to expect and need to be orientated to adult services before they transfer<br><br>**Theme:** Continuity of care<br>**Insight Statement(s):**<br>• Young people and families move from a very familiar team in children's services to the unknown in adult services<br>• Young people feel like they are bounced from pillar to post with no stability<br><br>**Theme:** Handover of information<br>**Insight Statement(s):**<br>• Young people and parents feel the onus is on them to repeat histories and pass on information to new health professionals<br><br>**Theme:** Growing the GP relationship<br>**Insight Statement(s):**<br>• GPs are expected to fill the role of the paediatrician in adult services<br>• Young people often have a limited relationship with their GP pre-transfer<br>• A trusting relationship with your GP is essential to ensure good communication<br><br>**Theme:** Housing<br>**Insight Statement(s):**<br>• Young people need information on housing options

					• Local authorities should include young people on housing lists at 18 years and ensure appropriate housing options, outside the family home, are available<br><br>**Theme:** Employment<br>**Insight Statement(s):**<br>• Young people need information on employment rights<br>• Young people need work experience so they can learn how to manage fatigue etc.<br>• There is a lack of employment/work experience opportunities<br><br>**Theme:** Community integration<br>**Insight Statement(s):**<br>• Young people need to have a purpose in the community<br>• Young people face transport and structural barriers to community integration
Workshop 1 & 2	Define	How Might We (d.school, 2018, p. 13)	"How might we" (HMW) questions are short questions that launch ideation. They're broad enough to include a wide range of solutions but narrow enough to impose helpful boundaries. Start with your design challenge and Point of View statement (see card #11).<br>Then break down the larger challenge into smaller actionable bits and ask questions that openup the solution space.	Insight statements were rephrased as questions by adding "How might we" to the beginning of the statement How might we (HMW) questions were shared with and reviewed by advisory group members and researchers (GL, MM, TK) over email.	Nineteen HMW questions were developed:<br>1.How might we improve knowledge and awareness about CP among healthcare professionals?<br>2.How might we inform young people about their CP and what aging with CP means?<br>3.How might we support young people to manage their CP in adulthood?<br>4.How might we ensure that young people are involved and engaged in the planning of their transition process?<br>5.How might we ensure that the transition process takes full account of the young person's views, needs and hopes for the future?<br>6.How might we give young people and their families or carers information about what to expect from the transition process and what support is available to them?<br>7.How might we ensure that information about the transition process and supports is accessible and easy to find for anyone who needs it?<br>8.How might we ensure that each young person approaching or entering the transition phase receives care and support that is appropriate to their age and stage<br>9.How might we support young people to develop independence to direct their healthcare and support throughout the transition process?<br>10.How might we build young people's confidence to make decisions and direct their own healthcare and support over time?

					11.How might we ensure young people's individual needs and perspectives on their healthcare are listened to?<br>12.How might we prepare young people for the changes in their role and responsibilities in managing their healthcare in adulthood?<br>13. How might we build a connection with adult services before they leave children's services?<br>14.How might we ensure greater continuity for young people transferring between children and adult services?<br>15.How might we facilitate the passage of information from children's to adult services?<br>16.How might we proactively engage primary care in transition planning?<br>17.How might we support young people to develop a supportive relationship with their GP?<br>18.How might we support young people's confidence in communicating with unfamiliar adult service providers?<br>19.How might we ensure that transition addresses all relevant outcomes including those related to education, employment, community inclusion, emotional wellbeing and independent living?
Workshop 1 & 2	Define	Brainstorm selection (d.school, 2018, p. 17)	Your brainstorm should generate many, wide-ranging ideas. That is the easy part. The hard part is selecting which ideas to act on. Brainstorm selection may be straightforward for some brainstorms (simply pick a few standout ideas), but selecting design solutions may take more deliberation.<br>Don't pick just one and don't settle on safe choices. Select a range of ideas to carry forward into prototyping, aiming to preserve the breadth of solutions generated by the group.<br><br>How to select brainstorm ideas:<br>Don't narrow down ideas too fast. An implausible idea may spark a useful or meaningful insight. Hang onto ideas that excite, amuse, or intrigue the team. Consider these three selection techniques.<br>Post-it voting.<br>Each team member gets three votes. The post-its with the most marks are selected.	To determine which HMW questions to focus on advisory group members were asked to rate the HMW questions from 1-19 in order of perceived importance using an online survey. The top ten HMW questions were used as the starting point for brainstorming solutions.	The top ten HMW questions were:<br>1. How might we give young people and their families information about the transition process and what support is available to them?<br>2. How might we ensure that the transition process takes full account of the young person's views, needs and hopes for the future?<br>3. How might we ensure that information about the transition process and supports is accessible and easy to find for anyone who needs it?<br>4. How might we ensure that transition addresses all relevant outcomes including those related to education, employment, community inclusion, emotional wellbeing and independent living?<br>5. How might we ensure young people's individual needs and perspectives on their healthcare are listened to?<br>6. How might we build a connection with adult services before young people leave children's services?<br>7. How might we ensure that young people are involved and engaged in the planning of their transition process?<br>8. How might we improve knowledge and awareness about CP among healthcare professionals?<br>9. How might we build young people's confidence to make decisions and direct their own care and support over time?<br>10. How might we support young people to develop

					independence, including independence to direct their care and support throughout the transition process?
Workshop 3	Ideate	Brainstorm rules (IDEO.org, 2015, p. 95)	We've all been in Brainstorms (p. 94) that went nowhere. At IDEO.org, the goal isn't a perfect idea, it's lots of ideas, collaboration, and openness to wild solutions. The last thing you want in a Brainstorm is someone who, instead of coming up with ideas, only talks about why the ones already mentioned won't work. Not only does that kill creativity, but it shifts the group's mind-set from a generative one to a critical one. The only way to get to good ideas is to have lots to choose from.<br><br>STEPS<br>01 Defer judgement. You never know where a good idea is going to come from. The key is to make everyone feel like they can say the idea on their mind and allow others to build on it.<br>02 Encourage wild ideas. Wild ideas can often give rise to creative leaps. When devising ideas that are wacky or out there, we tend to imagine what we want without the constraints of technology or materials.<br>03 Build on the ideas of others. Being positive and building on the ideas of others take some skill. In conversation, we try to use " yes, and…" instead of "but."<br>04 Stay focused on the topic. Try to keep the discussion on target, otherwise you may diverge beyond the scope of what you're trying to design for.<br>05 One conversation at a time. Your team is far more likely to build on an idea and make a creative leap if everyone is paying full attention.<br>06 Be visual. In Brainstorms we put our ideas on Post-its and then put them on a wall. Nothing gets an idea across faster than a sketch.<br>07 Go for quantity. Aim for as many new ideas as possible. In a good session, up to 100 ideas are generated in 60 minutes. Crank the ideas out quickly and build on the best ones.	We focused on three brainstorming rules<br>1. There are no bad ideas in a brainstorm<br>2.Feel free to build on the ideas of others<br>3. Go for Quantity	Brainstorming rules were discussed at the beginning of workshop three. Members were invited to add additional rules if they wished.

Workshop 3	Ideate	Brainstorming (d.school, 2018, p. 15)	Brainstorming conjures tons of ideas all at once. A brainstorm is a distinct segment of time when you amp up the generative part of your brain and turn down the evaluative part. The intention is to leverage the collective thinking of the group. Brainstorming can be used throughout the design process: to plan empathy work, to assess products and services, and to come up with design solutions.<br><br>How to brainstorm:<br>Your team's sole goal is to generate as many ideas as possible, without judgment. Gather in front of a whiteboard and spend 15 to 30 minutes in high engagement "brainstorm mode."<br>Be sure to capture every idea, regardless of your feelings about them. You can either assign a scribe to capture ideas as they're called out or go all-in, each person shares their ideas out loud and puts them on the board themselves. Either way, use post-its and stick them up quickly. You can use How Might We questions to launch a brainstorm (see card #13).	YPAG and PAG members brainstormed as many solutions as possible to the top ten HMW questions. Separate Miro boards were created for the YPAG and PAG. A section for each question was created on Miroboard. Each question had colour coded post it notes. Each idea in response to a HMW question was written on a post-it on Miro board.	345 post-its were generated by the YPAG and PAG in response to 10 HMW questions.

Workshop 3	Ideate	Bundle ideas(IDEO.org, 2015, p. 97)	Bundling Ideas takes you from strong individual concepts to solutions of substance. Think of it as a game of mix and match, with the end goal of putting the best parts of several ideas together to create more complex concepts. You've probably noticed that many ideas start to resemble each other—which is a good thing. Try different combinations; keep the best parts of some, get rid of the ones that aren't working, and consolidate your thinking into a few concepts you can start to share.<br>STEPS<br>01 You've got lots of drawings and ideas up on the wall, so now it's time to start moving them around and forming them into more complex solutions.<br>02 Start by clustering similar ideas into groups. Talk about the best elements of those clusters and combine them with other clusters.<br>03 Now, start building groupings out of the themes and patterns you've found. Focus on translating what you've heard into practice, rather than just identifying similar ideas.<br>04 Once you've got a few idea groupings, ask yourself how the best elements of your thinking might live in a system. Now you're moving from individual ideas to full-on solutions	Similar post it notes were bundled together into groups and patterns. Although young people and parents identified many similar solutions, some solutions were only identified by one group.	**YPAG and PAG**<br>1. Transition website<br>2. Designated transition coordinator who shares transition information<br>3. Transition booklet that outlines transition process and information<br>4. Videos that share transition experiences<br>5. Health professional education<br>6. Parent support network<br>7. Transition training programme for young people and parents<br>8. Peer support group for young people<br>9. Conference/ Webinar to provide transition information<br>10. Transition roadmap that provides transparent outline of process<br>11. Provide opportunities and skills to support emotional wellbeing<br>12. Health professionals should encourage autonomy by directing focus to young people in sessions<br>13. Annual review in adulthood to ensure needs are being met<br>14. Funding/resources must be adequate for successful transition<br>15. Provide opportunities to meet with adult team before transfer<br>16. Information content needed for transition<br>17. Work experience provision<br><br>**YPAG only**<br>1. Transition App<br>2. Contact point for emotional well-being<br>3. Newsletter for parents<br>4. Diary to note concerns and questions for appointment<br>5. Develop confidence and self-advocacy skills<br>6. Introduce and practice responsibilities for adult services before transfer<br>7.Parents need to step back to let young people try things themselves and build independence<br>8. Checklist of transition goals<br><br>**PAG only**<br>1. Podcast to share transition experience<br>2. Additional time in appointments to allow young people space to make decisions and ask questions<br>3. Defined age to begin transition process<br>4. Adult CP specialist centre or organisation<br>5. Copy of all correspondence provided to young person

					and parent<br>6. More transparency around funding options and allocation<br>7. Holistic/Person centred transition process<br>8. Database of people with CP to highlight and track need<br>9. Transition planning meeting with health professionals, parents and young person<br>10. Information evening/ fair for transition process<br>11. Handover process between child and adult health professionals<br>12. Better provision of living options<br>13. Directory of support persons<br>14. Map of available services
Workshop 3	Ideate	Reality check (Ideo, 2022, p. 54)	**Do a Reality Check**<br>So far, you have (hopefully) been developing your idea without giving much thought to the constraints you may face while attempting to realize it. It makes sense to now do a reality check: look at what's most important about your idea and find ways to evolve and develop it further.	Ideas were examined by the researchers (JR and JF) to determine their core value. Ideas were evolved by combining ideas with similar core values together.	•"Newsletter for parents" and "Transition booklet that outlines transition process and information" and "Transition roadmap that provides transparent outline of process" and "Information content needed for transition were combined to form "written informational support"<br>•"Conference/ Webinar to provide transition information" and "Information evening/ fair for transition process" were combined to form "conference or webinar"<br>•"Videos that share transition experiences" and "Podcast to share transition experience" were combined to form "digital stories of transition experience"<br>•"Contact point for emotional wellbeing" was amalgamated into a role for the "designated transition coordinator who shares transition information"<br>•"Diary to note concerns and questions for appointment" and "Checklist of transition goals" were amalgamated into "transition checklists and worksheets"

Workshop 3	Ideate	Describe idea (Ideo, 2022) p.55	Describe Your Idea<br>Once an idea has started to evolve, you may find it helpful to capture your thoughts in<br>a more structured format. Create a concept description. Consider it a repository for thoughts and questions rather than a finished piece.<br>Capture your thoughts<br>With your team, use a large sheet of paper to summarize<br>your idea. Use the following structure to describe its most important aspects:<br>» Choose a title for your idea<br>» Summarize your idea in a single sentence<br>» Describe how your idea would work<br>» Name the people it involves, both to build as well as<br>to use it<br>» Explain the needs and opportunities identified through field research<br>» Illustrate the value and benefit for each person involved<br>» List questions and challenges<br>Evolve your summary<br>Change and adjust your concept description continuously<br>as you prototype and iterate your idea. Keep<br>it in a place that is visible to all team members.<br>Before building	We summarised each idea in a single sentence and described how the idea responded to the transition needs identified.	Thirteen descriptions of ideas were created.<br>1.<br>**Idea title:** Map of available services<br>**Idea Summary:** An interactive national map of services for people with cerebral palsy to improve knowledge of and ability to access services.<br>**How will this idea impact the challenge**: We learned that finding out information about adult services or accessible facilities is challenging. Creating a map with a directory of services would help this.<br><br>2.<br>**Idea title:** Directory of support persons<br>**Idea Summary**: A directory of support persons/carers with profiles that list their interests, experience and hourly rate.<br>**How will this idea impact the challenge:** We learned that PA support is important for young adults. However, finding support people with special skills to work with people with CP can be challenging. The importance of working with someone you have common interests with was also highlighted. Creating a directory of support persons/carers who list their interests and special skills would allow young people and parents to choose suitable people to work with.<br><br>3.<br>**Idea title**: Transition website<br>**Idea Summary**: A transition website that acts as a centralized source of trusted and reliable information about the transition process.<br>**How will this idea impact the challenge:** We learned that information about the transition is difficult to find. A website would act as a centralized source of information that is easily accessible to a range of people.<br><br>4.<br>**Idea title:** Written informational support<br>**Idea Summary:** A written information source that can be given to young people and families to provide information about the transition process, for example, an information leaflet, transition packet or booklet.<br>**How will this idea impact the challenge:** We learned that 1) people may not know where to look or might not be engaged enough to go looking for information, 2) some families prefer a physical handout they can refer back to 3) service providers lack information resources to hand out. Providing a core document/leaflet/package to everyone entering the transition period would be beneficial to ensure

					information provision.<br><br>5.<br>**Idea title:** Conference or webinar<br>**Idea Summary:** A conference or webinar hosted in collaboration with young people and their families to provide information on the transition process.<br>**How will this idea impact the challenge**: We learned that young people and parents lack knowledge about the transition process. An online conference or webinar is one way of sharing information. The conference or webinar should be hosted in collaboration with young people and parents and include personal stories of transition.<br><br>6.<br>**Idea title**: Digital stories of transition experience<br>**Idea Summary:** Digital stories that capture personal experiences of transition.<br>**How will this idea impact the challenge**: We learned that young people and parents lack knowledge about the transition process and lack peers and role models to learn from. Creating videos of people's transition experiences would allow young people to learn from others who have gone through the process.<br><br>7.<br>**Idea title:** Parent support network<br>**Idea Summary:** A parent support group or network to connect parents with similar experiences. **How will this idea impact the challenge**: We learnt that parents have few opportunities to meet other parents with similar experiences. This is particularly true in adolescence and adulthood. Parents of young people who have gone through the transition could support and share information with parents who are just beginning the transition process.<br><br>8.<br>**Idea title**: Peer support group for young people<br>**Idea Summary:** A young person's peer support group or network to connect young people with similar experiences or connect young people with others who have gone through the transition process.<br>**How will this idea impact the challenge**: We learned that young people would value the opportunities to meet with other people who have been through the experience to support each other, share experiences and help each other

					problem-solve.<br>9.<br>**Idea title:** Transition checklists and worksheets<br>**Idea Summary:** Checklists and worksheets for young people to document what they want to discuss at their next appointment **How will this idea impact the challenge:** We learned that young people may not know what to ask and that there is an assumption that young people who don't ask questions are on top of it when this isn't always the case. Having a checklist that is standard for everyone would help this. We also learned that young people may struggle to remember concerns between appointments and having a space to document this may assist.<br><br>10.<br>**Idea title**: Transition App<br>**Idea Summary:** An app to support young people during transition. The app may include information on the transition process, a profile with transition goals and a place to document notes, concerns and questions to discuss in the next appointment<br>**How will this idea impact the challenge:** We learned that young people might need support with planning for the transition. Creating an app where they could record their goals and notes would be a user-friendly way to address this<br><br>11.<br>**Idea title**: Health professional education<br>**Idea Summary:** An educational programme for health care professionals to improve knowledge and awareness about CP and how to best support young people with CP during the transition.<br>**How will this idea impact the challenge:** We learned that service providers lack knowledge about CP, may have assumptions about CP and may not know how best to support young people with CP during the transition. Creating an educational resource for HCPs would help this.<br><br>12.<br>**Idea title**: Designated transition coordinator<br>**Idea Summary**: A health professional in a dedicated transition coordination role to support young people with their goals, advise on and facilitate the transition process.<br>**How will this idea impact the challenge:** We learned that young people and families don't know what to expect about the transition or how to navigate to adulthood. A designated transition worker with knowledge of the process

					could help young people plan their goals, advocate on behalf of the young person, act as an information source and provide reassurance.<br><br>13.<br>**Idea title:** A transition training programme for young people and parents<br>**Idea Summary:** A transition programme or course to empower young people with the knowledge and skills required to transition to adulthood. It may include modules about the transition process, emotional well-being and living options<br>**How will this idea impact the challenge:** We learned that young people and families feel unprepared for transition and that having a training programme that would cover life skills would be helpful

Workshop 3	Ideate	Design Principles (IDEO.org, 2015, p. 97)	Design Principles are the guardrails of your solution—quick, memorable recipes that will help keep further iterations consistent. These principles describe the most important elements of your solution and give integrity and form to what you're designing. Odds are, they will align with the themes you found earlier in the Ideation phase. You'll also find that they'll evolve as you design things, so don't be afraid to revise them. Keep them short and memorable, like, "Talk like people talk" or "Keep women at the centre of the business." Lower-level ideas like "The logo is blue" are not Design Principles.<br><br>STEPS<br>01 Look at your most important Post-its and what you came to in Finding Themes (p. 80) in particular.<br>02 Consider the core principles underpinning those themes. Frame these as positive statements that might tell you how and what to design.<br>Remember, Design Principles operate as a group, and it's likely that you'll need to identify several.<br>03 Look at the Design Principles you've come up with. Are they short and to the point? Do they describe just one idea? Try to avoid overly complicating them. If it feels like there are multiple ideas going on, break them into smaller parts.<br>04 Review your Design Principles and make sure they cover the key aspects of your solution. Modify any that don't.<br>05 Be ready to revise your Design Principles as you start to build prototypes and test your ideas. Some Design Principles won't reveal themselves until you've actually designed and tested something, but once you spot them they'll become essential.	Throughout the brainstorming, ideas from the advisory groups highlighted the need for accessible resources. For example, they highlighted the need for easy-read and jargon free information, consideration of individuals who are non-verbal, and individuals who may have hearing difficulties. We used this information to form an overarching design principle for any resources we would develop.	Design principle:<br>We will ensure that any resources developed are accessible.

Workshop 3	Ideate	2 x 2 matrix(d.school, 2018, p. 9)	A 2x2 matrix is a tool to scaffold information about users and your design space to reveal relationships. The hope is to uncover insights or areas to explore more deeply. A 2x2 matrix is also a great way to visually communicate a relationship you want to convey to others.<br><br>How to use a 2x2 matrix:<br>Draw a 2x2 matrix (x-axis and y-axis), pick a spectrum for each axis (opposites on either end), and plot items on the map. You can explore any group of things—products, motivations, users. You might place products on a matrix of perceived quality (low to high) versus use of natural materials (all-natural to all-synthetic). Where do groups form? Notice full or empty quadrants. Where does the assumed correlation break down? You may have to try various combinations of spectra to find one that's meaningful. Often the discussion spurred by filling in the matrix is more valuable than the map itself. You can also use a 2x2 matrix to create a competitive landscape. An empty quadrant may signal a market opportunity (or a very bad idea).	Advisory group members evaluated the thirteen ideas by a survey which rated each idea on a 5 point scale based on its feasibility and importance. We used a 2x2 Matrix as a decision support tool to determine which ideas to pursue and which to discard. Each axis represented a distinct dimension ranging from low to high importance and low to high achievability. Results from the YPAG and PAG were combined to determine the concepts that are achievable in the short term with potential for high impact.	The following ideas were rated as above average importance or achievability:<br><br>1. A transition website<br>2. Written informational support<br>3. Digital stories of transition experience<br>4. Transition checklists and worksheets<br>5. A transition app<br>6. Health professional education<br>7. Designated transition coordinator<br>8. A transition programme or course
Workshop 4 & 5	Prototype	Describe your concept (d.school, 2018, p. 33)	Many brainstorms end with a team realizing they don't fully understand the idea they selected to act on. The Describe Your Concept tool helps you distil down what resonated with the team from the original concept, and understand exactly what they need to create during prototyping<br><br>How to describe your concept:<br>Select an idea from your brainstorm and flesh out the concept while it's fresh in your team's mind. What would you name it? Who is it for? What does it accomplish for them? How does it accomplish this? Here, your team can really specify how your product or service does what it does. Make it your goal to articulate a single function that your concept performs in order to achieve the desired user impact.<br>*Pro tip: If your concept either 1) doesn't relate to your user or 2) doesn't bring you to a game-changing idea, don't worry, you've made a*	In workshop 4 and5 each of the eight ideas was brainstormed in detail to determine the user group, format and content. The research team translated insights gained during these discussions into eight resource prototypes. These resource prototypes were shared with an advisory group of health professionals with experience of working with young people with CP to obtain feedback and further information that would optimize the utility of the resource in practice	Resource prototypes are described in supplementary table 2.

			common mistake. Revisit your brainstorm and pick another idea that's more user-centric.		
Workshop 4 & 5	Prototype	Gut check(IDEO.org, 2015, p. 110)	The Ideation phase is about coming up with as many innovative ideas as possible, often with less emphasis on plausibility or implementation. At IDEO.org, this approach certainly leads to more creative thinking, but we also know that some of our more far-out ideas are probably better left on the drawing board. This Gut Check exercise can help you look at your ideas through a more critical lens and help you decide which ideas truly merit your efforts.<br><br>STEPS<br>01 Have a look at your most promising ideas and try to distil them down to their essences. For example, if your idea is about redesigning the patient experience in a health clinic, the core idea might be achieving more patient privacy.<br>02 Now, list all the constraints and barriers that stand in your way. Put them on Post-its and display them for everyone to see. Don't feel daunted if the list is long. Constraints make for great design!<br>03 This might be a great time to have a quick Brainstorm (p. 94) about how to evolve your idea within the constraints you just listed. How can you keep the core of your idea but push it so that it remains within your capabilities?<br>04 Don't be afraid of letting an idea go. The Gut Check is here to help you make the most promising ideas real.	We identified constraints as time and budget and narrowed to four prototypes	1. digital stories of transition experience<br>2. written informational support<br>3. a transition website<br>4. transition checklists and worksheets.

Table 2 Resource Prototypes

**Written informational support**	**Format**	**Content**	
**Purpose:**<br><br>• Reduce chance element of information sharing	• Leaflet/booklet<br>• Hosted on website<br>• Includes alternative accessible formats e.g. Easy Read	**Information about transition**<br><br>• What is transition<br>• When does transition happen<br>• How will it affect me	**Directories**<br><br>• Directory of further education options

• Prompt families to think about the transition process<br>• Act as a communication tool to begin transition conversation<br><br>**Users**<br>• Young people<br>• Parents		• What will happen going forward<br>• What services need to transition<br>• Where do young people transition to<br>**Information about adult services**<br>• How adult services work<br>• Differences between children's and adult services<br>• Introduction to new responsibilities in adult services<br>• What you should be prepared for in adult services<br>• How to access help and assistance in adulthood<br>• *Include caveat that service users check with local provider for further details as individual situations will vary*<br>**Cerebral palsy**<br>• Information about cerebral palsy<br>• Information on aging with cerebral palsy<br>**Glossary of terms related to transition**<br>• What is a hub<br>• What is a catchment area<br>• What is a multi-disciplinary team<br>• What is a profiling meeting<br>• What is an activity based centre<br>• What is New Directions<br>• What is a personal assistant and how can they support me<br>• List of relevant disability legislation and brief explanation of each Act	• Directory of accessible facilities, and changing places.<br>• Directory of mental health supports<br>• Directory of personal assistant supports<br>• Directory of adult services with service details including<br>o Location of service<br>o Age range catered for<br>o Disability types catered for<br>o Activities offered<br>o Staffing offered<br>o Respite attached<br>o Transport included<br>o Hours available<br>o *Level that programme is pitched at*<br>o *Care needs catered for to enable participation*<br>Rights and entitlements<br>• Information on entitlements<br>• Signposting to relevant sources of information and services e.g. citizens advise, revenue<br>Funding information<br>• Information on the system of funding and how funding negotiations work
**Transition checklists and worksheets**	**Format**	**Content**	
**Description**<br><br>A suite of documents that includes:<br>• A space where young people can document questions and concerns	• PDF hosted on website<br>• Printed paper materials<br>• Part of an app	**Checklists**<br><br>List of considerations for the transition process<br>• I have a contact point in adult services<br>• I know who my adult community team will be<br>• I know what equipment I need in adult services	

• A space to set transition goals, aspirations and monitor progress<br>• A checklist of areas and actions to consider during the transition process<br><br>**Purpose**<br><br>• Aid transition preparation<br>• Act as communication tool. Health professionals can view questions, concerns and goals and provide information and support<br>• Prompt young people and parents to think about the transition process<br>• Create accountability for the transition process by setting goals and monitoring progress<br>**Users**<br><br>• Young people<br>• Parents		• I am aware what equipment will transfer from child to adult services<br>• I know how to maintain my equipment<br>• I know what a key worker is and how they can support me<br>• I know what a personal assistant is and how they can support me<br>• I am aware of my rights and entitlements<br>• *I have received an individual family service plan*<br>• *I have discussed my family service plan*<br>Checklist of considerations if applying for funding package<br><br>• Transport<br>• Respite<br>Checklist of required documents for transition<br><br>• Medical reports<br>• Proof of catchment area<br>**Worksheet to document**<br>• Notes<br>• Worries about healthcare needs<br>• Questions or concerns for health care appointment<br><br>**Goal worksheet**<br>• Space to document young person's transition goals and aspirations<br>• Space to track and review goals
**Digital stories of transition experience**	**Format**	**Content**
**Purpose**<br><br>• Share learnings from people who have gone through transition<br>• Showcase peers and role models who are further ahead in the transition journey<br>• Provide information in an accessible bite sized format<br>• Prompt young people and parents to think about the transition process	**Participants**<br><br>• Real people talking about their experience is preferred to animated stories<br>• Young people may share their experiences<br>• Parents may share their young person's story on their behalf or share their own experience<br><br>• For education on cerebral palsy, a mixture of people	**About transition**<br><br>• Information about transition<br>• Successes and challenges of transition<br>• Emotions around transition (nervousness, letting go and associated emotions)<br>• Benefits of growing up<br>• Hopes and goals for the future<br>• *Consider questions like "What does transition mean to you"*<br>**About adult services**<br><br>• What adult services is like<br>• What getting a new adult health professional is like<br>• Responsibilities in adult services<br>• Experience of first visit to adult services

**Users**<br><br>• Parents<br>• Young people<br>• Family, siblings, grandparents<br>• Health professionals	with cerebral palsy and health professionals is acceptable<br>• Participants should be 16-25 years (Parent advisory group)<br>• Participants should include a wider age range to include adults with cerebral palsy at different life stages (Young person advisory group)<br>• *Including different ages and stages will offer a variety of perspectives. Including people who have just transitioned and people who are in their 30's will offer different insights (Health professional advisory group)*<br>• Include different representations of disability<br>• *Include people from different geographical regions. Transition experience will differ depending on where people are in the country*<br><br>**Length:**<br><br>• Mixture of short and long digital stories<br>• Vary length by the depth of the topic. Shorter length for emotive or intense topics.<br>• *Consider short individual digital story clips in TikTok style*<br>**Accessibility**<br><br>• Must be accessible to all.<br><br>• *Consider accessible formats for example closed*	• Differences between adult and children's services<br>• Video induction/introduction to adult services<br>• *Consider questions like "What was the main difference between child and adult services that you experienced?"*<br>**How to get ready**<br><br>• Tips for talking with the adult health professionals<br>• Gaining your own voice/becoming more independent<br>• How you can prepare, anything that helped you on your journey<br>• Advice for other parents:<br>o what helped<br>o how to ask for help<br>o how to find an advocate<br>o how to reach out for support<br>o avoiding burn out<br>o pursuing respite<br>o self-care<br>• *Consider topics like "what helped me was"*<br><br>• *Consider potential topics like "preparing my child for turning 18, what I wish I had known "*<br>**CP education**<br><br>• What is CP<br>**Becoming an adult/broader transition**<br><br>• Living options<br>• Relationship, LGBTQA, Sexual health<br>• Mental health, emotional well-being and self-care<br>• Working life with CP for example preparing a CV, disclosing a disability to an employer

	captioning, sign language interpreter<br>• Include option to filter digital story collection by topic so young people and parents can select the most relevant stories<br>• Digital story should contain all information content. Displaying links for information relevant to digital story after the video is acceptable<br>• Include option to contact people in the digital story with questions<br>Structure<br>• Blog style<br>• Each digital story should follow a format<br>• *Display the question asked and then the video of participants response*<br>• One person speaking for the duration of digital story<br>• Or multiple participants speaking on a topic, edited together to form a digital story<br>• Consider format of young adult talking to a younger adult<br>• *Consider template like "telling my story to my younger self"*	
**Transition Website**	**Format**	**Content**
Purpose<br>• Provide an accessible, centralised source of transition information	• Optimised for searching through simple Google search terms	**About transition**<br>• What does transition mean/What does transition entail

• Provide centralised repository of information specific to cerebral palsy<br><br>Users<br><br>• YPAG: Parents or young adult/late adolescence. Early to mid-adolescence may not engage<br>• PAG: Young adults and parents	• Linked on key disability sites<br>• Simple and accessible<br>• Engaging design<br>• Information mainly contained in digital stories rather than downloading documents	• Roadmap to transition: Who do I need to talk to, what do I need to do, how does transition work<br>• Preparing for adult services: Differences between child and adult services, what to expect in adult services; what a day in adult services is like<br>• Tips on how to talk to professionals in adult services<br>• Glossary- what is a hub, catchment area, MDT, activity based Centre<br>**Managing my healthcare**<br><br>• How I can manage my healthcare<br>• Information on being healthy<br>• CP specific information and advice about mental health<br>**Ageing and CP**<br><br>• What could I develop<br>• What am I trying to prevent<br>**Downloadable resources**<br><br>• Transition checklists<br>• Goal worksheets<br>• Question and notes worksheets<br>**Non-healthcare transition Resources & support (signposting)**<br><br>• Travel training<br>• Housing/Living options<br>• Personal assistants<br>• Respite information<br>• Employment/Work experience<br>• Independent living skills<br>• Further education options/schemes<br>• Advocacy<br>o How I can become an advocate<br>o Advocacy training<br>o Advocacy organisation<br>• Entitlements and allowances<br>• Directory of adult services options with service details<br>• Links to other organisations supporting exercise<br>Disability rights<br><br>• Links to relevant Citizen's information and Revenue<br>• Directory of mental health supports<br>• Links to disability organisations and what they can provide
**Transition App**	**Format**	**Content**

Purpose

Communication tool

- Promote communication between health professional and young person through discussion of concerns, questions and goals documented in app
- Promote communication between parent and young person about the transition
- Assist with relaying information and medical history by recording summary information in app
- Reduce reliance on parents to answer questions by facilitating access to key medical summary for the young person

Transition readiness

- Provide information to support transition process
- Stimulate young person's interest in transition
- Prompt young people and parents to think about considerations for transition

Transition planning

- Prompt young people and parents to begin planning what they want from the transition through recording transition goals
- Create accountability by recording transition goals and aspirations

---

- Accessible, young person should be able to use App independently
- Easy to use, refined to most important points
- Headings to indicate different sections
- Colour coding of different sections
- Consider security- avoid overly sensitive medical information
- Flexible to allow basic interaction or more in depth interaction depending on user's need
- More fun, less informative. May contain link to website for further information
- *App should be filled with life, and interactive content. If it's just a series of documents it won't have any longevity*

My profile

- My goals/wants/wish list for transition.
    - Include a list of goal headings for example leisure, education
- Reason for visit today
- Concerns or questions I want to discuss at my appointment
- My notes
- Transition discussion and decision log

Health record/summary

- Statement of need
- Practical things I need for adult services, for example equipment
- Digital health passport
- Key points summary
- Snapshot/overall picture of who I am
- List of medications and calculations
- Clinical contacts, in case of emergency contacts

Information

- Digital stories to provide information
- Skills on managing emotions
- Transition checklists

Experience sharing

- Social hub that allows young people to communicate with their peers
- Digital stories to share experiences
- *If there is a social aspect it would need to be moderated and managed*

Expert consulting

- Frequently asked questions section
- Include feature to allow young person to digitally share content in the app with health professional

• Promote young person involvement and partnership in their transition process<br>**Users**<br>• Young people from early adolescence<br>• Parent can view app to discuss content as a family, with young person's permission<br>• Parent can use app on young person's behalf if they are not able to, with young person's permission<br>• Health professional can view concerns, questions, goals and medical summary with young person's permission		
**Educational programme for health professionals**	**Format**	**Content**
**Purpose**<br>• To assist health professional to support young people and families during the transition<br>• To improve knowledge and awareness about cerebral palsy in adult services<br>• To correct assumptions about cerebral palsy<br>• To improve accessibility of appointments in general medical settings by increasing awareness of cerebral palsy<br>**Users**<br>• GPs<br>• Support people/personal assistants/Carers<br>• Educational professionals	• Training module in undergraduate training<br>• Rotation/placement in cerebral palsy in undergraduate training<br>• Training module for qualified health professionals<br>• Conference for health professionals<br>• Educational video for health professionals<br>• People with cerebral palsy should present part of training<br>• *More disability education and awareness is being incorporated at undergraduate level*<br>• *Training module is likely to have a limited uptake among*	**Education on cerebral palsy in general**<br>• Education on impairments, severities and complex need<br>• Adjustments and support to make adult appointments accessible<br>**Ageing and cerebral palsy**<br>• Physical changes associated with ageing<br>• Additional health checks required for example bone scans<br>• *Education on cerebral palsy is very broad. Aging with cerebral palsy and the issues that arise would be more useful*<br>**Communication skills**<br>• How to communicate with young people at different stages of transition<br>• How to communicate in an age appropriate way and not talk down to young people<br>• How to talk to people with different impairments<br>• Learning to give young people time, space and understanding in appointments so they feel confident to speak up

• Adult healthcare providers who work in hospital setting<br>• Mental health professionals<br>• Employers<br>• Dental care professionals	*health professionals with a limited caseload of people with cerebral palsy. Online information on area specific supports, that can be accessed quickly is more likely to be helpful*<br>• *Include an educational video about what cerebral palsy is as a starting point*<br>• *Consider an E-learning module or multi-disciplinary course*	• How to use inclusive language<br>• How to provide family-centred care where appropriate in adult services<br>• How to support autonomy<br>• Improve understanding of level of support needed at each stage of transition<br>• Improve understanding of expectations for independence and provide appropriate levels of support to promote independence<br>• How to provide choices to young person<br>• Improve understanding of how to support mental health and emotional well-being<br>• *How to support self-efficacy for all young people*<br>• *Consider training on communication skills e.g. How do we talk to young people when we talk about transition?*
**Designated transition coordinator**	**Format**	**Content**
**Purpose**<br>• Improve understanding of transition process for young people and parents<br>• Support young people and families to navigate to adult services<br><br>**Users**<br>• Young people<br>• Parents<br>• Allow flexibility for health professional in dedicated transition role to work with young person individually or with young person and parents	**Dedicated role**<br>• Dedicated role focused on transition<br>• Single person in the role. Multiple people may lead to inaction or mixed messages<br>• Dedicated role allows for cumulative building of transition knowledge<br>• Person in the dedicated role should be transparently identified to young person and parents<br>• *Value in having one person with more knowledge and responsibility to help other professionals and act as single point of contact, however no one person will have the answer to everything*<br>• *Where single person is responsible, delivery of transition can be ad hoc. Role of designated transition coordinator could be to ensure oversight of process,*	**Advocate**<br>• Act as an objective advocate to act on the young person or family's behalf<br>• Support families when they feel they aren't being listened to<br>• Contact point<br>• Act as identified point of contact<br>• Someone you can call when you have questions<br>**Liaison/coordinator**<br>• Bring you on the journey<br>• Ensure things are put in place<br>• Ensure you know who your health professionals are<br>• Identify additional supports required e.g. psychological support<br>• Provide introduction to adult services<br>• Liaise with adult services on family's behalf<br>• Identify contact point(s) for family in adult services<br>• Put young people and families in contact with services<br>• Assist coordination with adult hospitals<br>• Assist with coordination of holistic services e.g. employment, social activities<br>• Ensure information/files transfer to adult services<br>• Ensure equipment transfers to adult services<br>• Pass on requirements to adult services<br>**Aid transition preparation**<br>• Help young person prepare for transition<br>• Inform young person on what to expect during transition<br>• Provide oversight of the transition process<br>• Guide young person and family through transition process

	ensure transition goals are met and all information and options are provided<br>**Ideal professional**<br>• Social worker<br>• Psychologist<br>• With appropriate experience and training other health professionals would be suitable<br>• Transition worker can link with other professionals for advice and information<br>• *Progressing Disability Services is moving away from discipline specific roles.*<br>• *The key worker from health and social care professions may be the most appropriate person for this role.*<br>• *Access to social worker to fulfil dedicated transition role might be difficult in different geographical regions*<br>**Stage of transition they assist with**<br>• Role should cover both paediatric and adult<br>• Consider two equivalent roles. Paediatric transition worker transfers person to adult equivalent<br>• *Ideally the role should move back and forward across the divide to work with the person who is transitioning until their early 20's, however young people currently transition at 18 years*<br>• *Working with young people until they are in their 20's*	**Information source**<br>• Give you the information and tools that you need<br>• Signpost to information sources<br>• Answer questions<br>• Highlight areas that families should be aware of and consider<br>• Provide information on wider transition options (e.g. employment, housing)<br>• Provide comprehensive information<br>• Provide basic information (e.g. will therapy continue, where will therapy be located, where to go for equipment)<br>• Provide direction on what to do for transition<br>• Provide an honest picture of what the transition options are and what they include (e.g. transport)<br>• Provide information on what is available and not available<br>**Establish goals**<br>• Develop individual transition plan<br>• Develop transition goals<br>• Support aspirations for adult services and the future<br>• Ensure young person's views/needs/hopes are considered as part of transition process

	provides flexibility to address topics they weren't ready to discuss at 18<br>**Relationship and rapport**<br>• A known health professional would be beneficial due to an established relationship and rapport<br>• A new health professional in this role may be beneficial as they haven't known the young person from childhood and may treat you in a more age appropriate way<br>• If the person is unknown offering 1:1 support initially to build relationship and trust important<br>• *There is merit in professional knowing the young person, their family needs, complexities and circumstances.*<br>• *If role is based in an organization and young person transitions to another organization need to consider if person in dedicated role can follow up when they leave paediatrics*<br>**Communication approach**<br>• Flexible communication based on need, continuous input may not be required<br>• Different communication options e.g. in person, email, phone, virtual depending on each person's needs/wants and available resources	
**Transition programme or course**	**Format**	**Content**

**Purpose**<br><br>• To prepare young people and parents for transition<br>• To empower young people and parents with the knowledge and skills needed to transition to adulthood<br>• To prepare young people emotionally for the transition<br><br><br>**Users**<br><br>• Young people<br>• Parents<br>• Targeting both young people and parents will ensure transition is talked about and reinforced at home<br>• *Targeting both young person and parent will lead to a stronger output*	**Programme should incorporate group format**<br><br>• Group format includes social element and allows young people to work with peers<br>• Include separate groups for young people and parents to allow young people to have their say and ask questions<br>• Joint parent and young person group may be appropriate where parent is communication partner for the young person, however Individual communication styles (e.g. non-verbal, assistive and augmentative communication users) should be facilitated<br>• Parent and young person group could run at the same time with each group in a different room. This would allow autonomy but parental support is close by if needed<br>• Where support is required a young person could attend with a sibling close to their own age<br>**Programme should be professionally led**<br><br>• Different professions may lead each session based on their knowledge/expertise/subject area<br>• In person format preferred to online format. It is easier to share your worries and concerns in person.	Module on the programme may include<br><br>• Knowledge about CP<br>• Increasing transition readiness<br>○ What is transition<br>○ Differences in adult services<br>○ Establishing goals and hopes for the future<br>• Developing autonomy and independence<br>• Building self-advocacy skills<br>• Developing confidence<br>• Managing emotional well being<br>• Managing physical health<br>• How to maintain/develop social networks as you transition<br>• Developing self-research skills<br>• Ageing and CP<br>• Vocation, employment, work experience options<br>• Individualized budget options<br>• Living options<br>• Community inclusion<br>• Relationships

	- However, online may suit those not confident enough to attend in person *Virtual could be easier rollout. Virtual route would allow more opportunities to run past the age of transition and do further follow up.* **When programme should be delivered** - Programme could be delivered in the evening, over a number of weeks. - Parent programme could be delivered through evening weekend course/conference - Programme could be included as part of a transition year, be covered in school or as a QQI module with support from educational professionals *Having an adolescent strand could target young people that are in their local school in the community but who still need to understand the path forward and build advocacy skills* **Information delivery** - Introducing all information at once may overwhelm. Topics should be split into different sessions - Programme should include core transition modules that are covered by all participants - Other optional modules should be available based on need - Some modules might be more appropriate when you	

	have transitioned (e.g. housing). Include option to revisit this information.<br>• *A lot of the topics like living options might only be for people who are aged 18 years and older*	
Italic text indicates additional comments from health professional advisory groups		

Table 3 Archived recommendations

Recommendation	Learnings	Design stage
Health systems recommendations		
Adequate staffing needed for transition	• There is a lack of staff on the ground<br>• Redeployment due to COVID worsened staff availability<br>• Staffing affects transition	Workshop 1 & 2 “Story share and capture”
Adequate funding needed for transition	•Funding/resources must be adequate for successful transition	Workshop 3 “Brainstorm”
Adequate funding needed to support adults’ aspirations	• Sufficient funding needs to be in place to support adult aspirations	Workshop 1 & 2 “Story share and capture”
More transparency needed around funding options and allocation	•Parents report limited transparency about available funding and where funding is allocated	Workshop 3 “Brainstorm”
Care staff should be valued	• Need to recognise the value of the work care staff do<br>• Need to fund care staff adequately and provide training	Workshop 1 & 2 “Story share and capture”
Better interagency working is needed	• Lack of joined up thinking<br>• No one takes responsibility for transition<br>• Lack of communication between professionals<br>• Health service and education department should liaise about transition earlier	Workshop 1 & 2 “Story share and capture”
Database of people with CP to highlight and track need	• Need process & platform to collect data on who is transitioning, who requires disability services and collect data about upcoming need<br>• Need accurate data/ registers to show gaps in service delivery so they can be addressed	Workshop 3 “Brainstorm”
Better provision of living options	•Independent living should be termed living options to reflect need for different models e.g. single occupancy, shared housing<br>•Counting young people on the housing list should be prioritised<br>•Living outside family home needs to be resourced sufficiently	Workshop 3 “Brainstorm”
Recommendations for adult services		

Recommendation	Learnings	Design stage
Reassessment and reviews should be accessible and timely in adult services	• Cerebral palsy may change as you get older. You may not get access to services when leaving children's services because of your level at transfer<br>• Need reassessment in adulthood so you are not on your own<br>• Check in and reassessment needed, as things may change as you age	Workshop 1 & 2 "Story share and capture"
	•Annual review in adulthood to ensure needs are being met	Workshop 3 "Brainstorm"
Healthcare in adulthood should be centralised	• Need to reduce fragmentation of services<br>• Everything should be in one place<br>• You should be able to see two or three professionals, on the same day, under the same roof	Workshop 1 & 2 "Story share and capture"
Healthcare in adulthood should be community centred	• Adults services should be community centred, in primary care	Workshop 1 & 2 "Story share and capture"
Healthcare should follow a life needs model	• Kids with CP become adults - need a lifespan model<br>• Need to look to the future from an early age<br>• Equipment should follow you rather than having to reapply in adulthood<br>• CP is for life, therapy access should be too. People shouldn't be expected to use private healthcare	Workshop 1 & 2 "Story share and capture"
Adult CP specialist centre or organisation	• Need adult hospital or service that addresses CP issues and has specialist knowledge of CP<br>• Need specialist institute or organisation for CP	Workshop 3 "Brainstorm"
Recommendations for transition services		
Recommendation	Learnings	Design stage
Age of transition	• Planning for adulthood should have a set age.<br>• It should start when you go into the senior end of school<br>• It should start before you transfer<br>• When domiciliary payment ends transition planning could begin	Workshop 1 & 2 "Story share and capture"
	Need defined age to begin transition process	Workshop 3 "Brainstorm"
Transition planning meeting with health professionals, parents and young person	•Include young people in planning meetings<br>•Consider combining educational and clinical transition plans<br>•Review meeting needed before discharge to assess needs and make referrals<br>•Series of meetings needed so families have time to plan	Workshop 3 "Brainstorm"
Handover process between child and adult health professionals	•Handover needed between staff to share knowledge/experience<br>•Handover meeting with both teams<br>•Period of co-working between teams would help ease transition<br>•Joint consultation to share information about young person between paediatric and adult health professionals	Workshop 3 "Brainstorm"

Provide opportunities to meet with adult team before transfer	•Establish adult service in advance of young person leaving paediatric services<br>•Facilitate visits/introductory meeting before transfer<br>•Facilitate induction to the new service (e.g., parking, timetable, staff, access, toilets)	Workshop 3 "Brainstorm"
Provide opportunities and skills to support emotional wellbeing	•Provide opportunities for young people to speak to others about how their CP makes them feel<br>•Provide access to professionals to discuss emotions and address skills to manage emotional well-being<br>•Provide skills on managing emotions<br>•Provide links/contact details to helplines with people trained around supporting emotional wellbeing<br>•Supporting emotional wellbeing should begin early and should be checked during transition because it's a vulnerable phase	Workshop 3 "Brainstorm"
Work experience provision	•Limited work experience opportunities<br>•Accessing work experience is challenging<br>•Transport barriers in community create barriers to employment<br>•Training needed on managing fatigue, employment discrimination and disclosing a disability to employers	Workshop 3 "Brainstorm"
Holistic/ Person centred transition process	•Use inclusive language<br>•Discussion currently focused on needs rather than wants<br>•Views, wants and hopes should be considered along with needs<br>•Addressing and planning for all holistic needs of young person should be considered during transition	Workshop 3 "Brainstorm"
Additional time in appointments to allow young people space to make decisions and ask questions	•When young people are rushed, parents jump in to make sure nothing is missed<br>•Giving young people more time in appointments will facilitate them asking questions and being more independent	Workshop 3 "Brainstorm"
Health professional should encourage autonomy by directing focus to young people	•Health care professionals sometimes speak more to parents than young people<br>•Including young people in the conversations and directing questions to them will help develop their independence<br>•Ask young person at every appointment do you want to answer the questions<br>•Start from a young age, don't just wait until transition	Workshop 3 "Brainstorm"
Parents need to step back to let young people try things themselves and build independence	•Parents need to let young people develop independence rather than doing everything for them<br>•Allowing young people to try things out and make mistakes is important<br>•Parents can create space for young person to give their opinions during appointments	Workshop 3 "Brainstorm"
Copy of all correspondence provided to young person and parent	•Address correspondence to young people<br>•CC family into correspondence, referrals and handovers	Workshop 3 "Brainstorm"

	•Provide copy of letters from paediatric healthcare service to adult healthcare service to young person and parent	
Develop confidence and self-advocacy skills	•Professionals need to be understanding that becoming confident is a process and requires time.<br>•Support should be removed gradually to grow confidence<br>•Self-advocacy is key to ensure health professionals listen to individual needs<br>•Improving condition specific knowledge needed to develop confidence and advocacy<br>•Training needed to build independence and autonomy including how to relay information to health professionals in school or groups	Workshop 3 "Brainstorm"
Introduce and practice responsibilities for adult services before transfer	•Need awareness and training on responsibilities before you leave children's services<br>•Need information and preparation for the differences and expectations in adult services	Workshop 3 "Brainstorm"

Table 4 Comments recorded in responses to 10 HMW questions

Q1	How might we give young people and their families' information about the transition process and what support is available to them?
YPAG	YouTube video information about the transition process
	Health service should run a conference about transition process and give information to parents and young people
	Hear from young people who have gone through the transition process at the conference
	Conference is a good option for parents and people who are not tech savvy
	App for young people - making it user friendly for young people
	Webinar for young people
	Newsletter for parents – sent via post - could include a link to website
	Online information
	Videos with young people who have gone through process available on an app
	Groups of older people that you can reach out to

	Start with basic information about transition and provide links to further information
	Have colour-coded different sections in app for different transition information
	Include information about adult services (e.g. role of keyworker)
	Videos about transition need to be accessible to everyone including people that can't read well/ who lip read/who use sign language/who are non-verbal
	Need to use real people in any resource rather than cartoons or animations
	Website (for parents)
	Have a question box on website
	Dedicated key worker just for transition who meets the young person informally from 2-3 years before transfer- talks about the young person's hopes/dreams, what they want from adult services)
	Key worker for parents and young person
	Key worker's only focus should be transition
	Key worker there when needed - but may not need continuous input
	Key worker needs to be 1:1 for young person so they can build a relationship
	Key worker needs to have knowledge of wider transition (e.g. employment. housing)
	Sessions to build independence and autonomy - and then move to sessions on how to identify resources
	Social groups on social media - have online support (identifying groups already out there)
	Need multiple information options for people
	Starting to create own timetable before you leave skill (taking on responsibility before you leave children's) - skills taught in school - include example adult services timetable
	Helpline for information
	Lack of transparency about entitlements for people with CP/not broadcast
	Transition to adulthood group that contains general information and core modules on transition would be a good idea
PAG	Need information relevant to physical disability including medical needs
	Need location specific information
	Institute/organisation for CP which focuses on CP
	CP issues at centre

	Reference booklet that includes information - need to include all information (e.g. from Citizen's advice, schools, GPs)
	Person/group to signpost to who has responsibility and knowledge of whole transition area
	Social media
	Podcasts (more accessible) - include successful transitions stories
	Job fairs/information fairs for transition process - need to include physical disability for all levels of impairment
	National information day online
	Information day/evening online that includes general information and more specific information
	Health service should have a young person's disability group
	Collect data on who's requiring disability services to keep track of need and highlight time to provide information. Could be linked with domiciliary care (age 16)
	Have key person with knowledge of CP to co-ordinate care in adult service/hospital
	Web page
	Parent word of mouth
	Network of parents of people with CP (limited numbers of parents with same experience)
	Need to link with parents of children with similar needs
	Create social opportunities for parents to share knowledge
	Conference - focus on CP and not just medical condition (e.g. CP and epilepsy)
	Education/Training/course on transition, in person, over a weekend (provides information and social interaction)
	Young person education/training
	Education/training online for parents
	Peer support among parents to share experience/provide support and not just information (online or in-person)
	Parents of older children/adults speaking to parents of younger children
	Public health nurses to share information /know where to go for information
	Documenting local guide to information
	Handover between staff to share knowledge/experience

	Physio to share information (physios are a link to all people with CP)
	Planning review depending on needs/ document of changing need and link to relevant professional
	People need a list of what is involved in all areas of transition
	Need a roadmap with information about what to expect as adults..
	Need to know generally what's out there
	Need a roadmap to show the path after children's services, don't know what is next
Q2	How might we ensure that the transition process takes full account of the young person's views, needs and hopes for the future?
YPAG	Starting to take on responsibility before you leave children's services- skills could be taught in school - include example adult services timetable
	Develop timetable and goals for adult services with teacher/SNA
	Share goal timetable with key worker so they know what the person's aspirations are
	Support needs to be offered to everyone regardless of how they appear (i.e. if they appear on top of it)
	Discuss goals with key worker
	Need a process to check in on everyone during transition
	Communication device/communication board - make sure vocabulary about transition/aspirations is in the communication system
	Need to have a dedicated role for someone to deal with transition and ensures young people's views/needs/hopes are considered
	Person who deals with transition needs to know about all areas of transition including employment, social participation - have an idea of different options
	Young person creates a profile with goals on the app - key worker can communicate with them through that - can follow-up goals over email if not in person
	Have options for meeting with a key worker - e.g. in person, email, phone - depending on each person's needs/wants
	Have one key worker to support transition rather than training multiple people - if multiple people have the role no-one might do anything or they'll give mixed messages
	Need someone professional to fight your corner
	1:1 transition key worker - not someone from outside, someone from in the service that knows you and has a rapport with you and can advocate for you. To have someone to advocate with you would be less worrying and anxious
PAG	Money/lack of funding - can't implement a plan even if there is one
	Need to be realistic when planning - no point in promising if it can't be implemented
	Transparency about what funding is available and where it is allocated

	Need review process to ensure that service is being delivered (e.g. if young person changes mind)
	Direct payment to young person/family
	Young person entitled to independent living even if living at home/family - resources should be in place to support this
	Holistic needs of young person - addressing all needs and planning for all of these during the transition process - need to include employment, socializing
	Need someone to co-ordinate holistic needs of young person - includes employment, socializing, independent living
	Identify other options provided internationally (e.g. Canada, UK, Scandinavia)
	Flexibility by organisations about how they operate schemes (e.g. leasing car rather than having to buy one)
	Mapping accessible services (changing services, parks, shopping centres)
	Need infrastructure in place to support needs/hopes - unfair to ask for young people and parents' views if you can't provide them
	Need staff/professionals to be realistic about what they can provide and signpost to other organisations that can provide alternatives
	Discussion currently focused on needs rather than wants. Include young person's views/wants in the conversation along with needs - during meeting with young person, family, teacher/professional, adult service provider/co-ordinater
	Services co-operate with each other to address the needs/wants of young person - rather than creating barriers
	Social media or podcasts
	Views and hopes need to be considered along with needs
Q3	How might we ensure that information about the transition process and supports is accessible and easy to find for anyone who needs it?
YPAG	App
	YouTube video
	Send out a leaflet to all people who are going to start transition
	Create a “transition package" to send to all people who are starting the process
	Use ads to target young people e.g. on social media
	Send link to the young person's email or via WhatsApp
	Send link with information to parent's email
	Include dedicated key worker's name and contact details in a link with videos about transition
	Didn't know about [service name]'s adult website when in children's services - need to be told about it much sooner

	Need to give the information to –parents, young people probably won't look up websites for it until older
	Involve parents in social groups/groups with adults with CP talking about their experience, if young people don't want to be part of it
	Have work experience option - transition in adulthood first before person has to look for a job/decides about a job
	Groups with older people with CP to hear about their experience
	Speak to older people who may be a role model - important for parents and young people
	Older person to speak to with advice/information about what they did/the support they found or used
	Need to use language about CP and speak to people with CP to learn about their CP. Parents mightn't know
	Need to speak to people with similar experience - e.g. non-verbal
	Roadmap of the rough stages of transition and what it looks like. Could be in video or web format
	Directory of adult services is essential. When you're not in the know, it's very difficult to find information on adult services
	Need to know where to go to for information about entitlements
	Road map - video/other formats for people who can't read
	Everyone should get a core document of key points of information
	Need to think about accessibility issues for others
	Accessible for all young people including non-verbal and with hearing impairment
PAG	Put information in all organizations (anywhere that comes up when you search for disability)
	Paper
	Online
	Citizens advice bureau
	Revenue for entitlements information
	Physio
	Paediatrician (sometimes for annual review)
	Word of mouth between parents
	Create opportunities for parents to meet to share information

	Directory of carers/personal assistants/other support - age appropriate, similar interests
	Through carers if they have an interest and are paid/valued enough
	Important that information is accessible to everyone
	Doing research and finding information maybe even more difficult for those where English is a second language
	People need skill development to search for information
Q4	How might we ensure that transition addresses all relevant outcomes including those related to education, employment, community inclusion, emotional wellbeing and independent living?
YPAG	Change the term "independent living"
	Need to provide shared housing options for people who don't want to live independently (i.e. alone)
	Have an option for single occupancy and shared housing (depending on person's choice)
	Provide work experience for people with disabilities - (new directions encourages you to get employment in your local community - if council doesn't offer work experience they aren't supporting people to get work in local community)
	Government departments should be incentivized to give people with CP work experience
	Need to start supporting emotional wellbeing in childhood - transition is a vulnerable phase - need to check in on young people then
	Need to train people to support emotional wellbeing
	Broader issue of lack of resources to support emotional wellbeing
	Fear among employers to employ someone with a disability
	Getting employment broadens views and builds confidence
	Change how health professionals talk to parents and parents talk to young people about disability - broaden views, change language
	Change how wider society views people with disabilities, language around disability
	Increase pay for personal assistants - attract people into system if better pay/value them more
	Provide skills on managing emotions (include in app)
	Provide links/contact details to helplines with people trained around supporting emotional wellbeing
	Coaching on whether you should disclose disability in interviews
	Work experience for people with disabilities needed - lack of opportunities/get told they don't take people for work experience

	Young people need to get on the housing list to make sure they are counted for housing
	Housing would be really important, so you're not left to find out things yourself
	Limited options for living outside the home. Unsuitable housing offered. Need more options around shared housing for people with CP - single occupancy model good for some people. Need shared housing model for people who need 24 hour care. Not nursing home.
	Need information on what employment discrimination looks like, so you can recognize when it's happening
	Community has to be willing to give work experience
	Getting experience in what a full work day is like and how to manage fatigue important
	Access to transport important for integration in community
	Structural barriers in the community to transport e.g. dipped curves limited ability to integrate in the community and socialize as you need
	Limited understanding of barriers to transport e.g. driving lessons
PAG	Professionally led group to discuss emotions and address skills to manage emotional well being
	Included in the literature that's given to people during the transition process
	Mention all of these areas during the transition process
	Mention all of these in relation to all severities/types of impairment
	Need to establish what is meant by these terms
	Establish meaning of independent living
	Living options for people with physical disability - more options/different models for ID but not for physical disability
	The wording of independent living needs to change to be applicable to the vast majority of people with disability - needs to be “living options”
	Need to expand the meaning of employment - "meaningful..." have an occupation/purpose - it may not be paid
	Provide opportunities for socializing
	Transition year for mainstream school - peer support group/match with peers to provide opportunities to create friendships
	Need opportunities to speak to others to talk through how their disability makes them feel/the impact of their disability on family - speak to a professional
	All people at 18 years of age should be on a housing list. Local authorities/councils would then know how many people need housing (or specialized housing). Numbers who opt out will be relatively small.
	Allow you to start pushing for supports (e.g. housing) early on because so you can see the needs coming down the line.

	It's only in crisis that there are residential options given
	There should be independent living options outside the family home for everyone
	Allows people to have a purpose within the community.
	Introduce transition year into special education needs school - build life skills, confidence - skills beyond education.
	A programme at 16 years would identify future needs at 18 years - give heads up about what will be needed to support this young person
	All "life skills training" info/training should be incorporated into a "transition year" programme - addressing occupational/housing/educational/relationships training. Everything within the life skills training subtheme will make the young person feel more "adult". Everyone can have some role (paid or not). Gives people adult skills. Include driving skills within this - direct to organizations that can support
	People in mainstream school could access transition year - but needs to be tailored to the person
	Also use as a platform to collect data about upcoming needs - e.g. people who will be needing housing
Q5	How might we ensure young people's individual needs and perspectives on their healthcare are listened to?
YPAG	Health service sends out a health report (by email or post) - ask about your health - ongoing questionnaire every 2 years throughout your life
	Post questionnaire to young person before the appointment to get their views beforehand
	Put worries into an app about their healthcare needs - health professionals will have to ask young person rather than parent because young person has written down their concerns
	Have a place for young person to take notes on an app
	Equipment assessments in adulthood need to be done on a yearly basis at home and in day service
	Screen people about overall health (physical health) - ensure their needs are met - young person may not know their needs - have a checklist - know if to refer for further assessment
	Young people with CP in conjunction with Health Service run a CP talk/conference on living with CP - through the different ages
	Young person needs confidence to know that they know their condition best/and stand their ground/ learn to self-advocate
	Write down concerns in advance so you know what you want to say in appointment - have a medical diary to note concerns
	Being sick of not being listened to makes you stand up for yourself
	Self-advocacy is key – people do not listen to individual needs
	Advocacy allows you to speak up and be a voice for others,
	When you suddenly have to advocate your confidence increases

PAG	Standard process that health professional asks all young people at every appointment do you want to answer the questions
	Disability specific service that allows extra time to address needs of young person, refer to others
	Specialist knowledge of CP among doctors and health professionals
	Doctors/health professionals need skills to talk to young person
	Young person is asked do you want to be the person that answers the questions - give them the choice - parent/mum sits back and interjects if something is incorrect or omitted
	Module in doctors/health professionals training
	Support with advocacy as well as services.
	Young people need to learn to relay information between service/school and families.
	Need to support young adults with time and understanding to make their feelings known
Q6	How might we build a connection with adult services before young people leave children's services?
YPAG	Build self-advocacy/ need knowledge about your condition to hit the ground running in adult services
	Resources might prevent services from doing anything
	Doctors (Paediatricians) in children's services should be invited to meeting in adult service - to share information about young person - Paediatrician to share knowledge about CP with adult doctor - joint consultation
	Key worker should be involved in joint consultation
	Health service need to create a transition department - person with CP works with transition department to identify areas to focus on transition (e.g. appointments that needs to continue)
	Transition department in Health Service (only deals with transition period) includes a person with CP who has gone through the experience
	Transition department includes school to adult services, employment opportunities, and health services
	Electronic health records. Some places have integrated records
	Young people should be able to access to own records
	Have someone in adult services explain how it works – e.g. expected to go by yourself without parents.
	Having a point of contact important in adult services is important
PAG	Establish adult service (education, day service) well before the person leaves the paediatric service
	Have visits with the adult service (e.g. school, day service) before the person goes

	Letter from paediatric healthcare service to adult healthcare service - provide copy to young person and parent
	Introductory meeting with adult healthcare service before they transfer - share notes
	Induction to the new service (e.g., parking, access, toilets)
	Have a person in the new hospital show you around/induction to show where different departments are
	School - need to provide introductory week. Like work experience. Induct to building, timetable, breaks, toilets, staff.
	Induction week in school - gives staff opportunity to get to know person and what adaptations may be needed
	Provide review of adult service/school and opportunity to change
	MDT meeting- keyworker good person to speak to initially. Health service representative should check in as an independent observer/provides audit or review of funding
	Period of co-working between teams would help ease worry
	Integrate patient records
	Need someone with broader oversight of the process to organize transition
	Need more social worker support for transition, as a point of contact to coordinate
	Want someone to hold your hand through the process and say they'll help you
Q7	How might we ensure that young people are involved and engaged in the planning of their transition process?
YPAG	Key to deal with young person/talking to the young person directly - parents may be in room but need to direct conversation to young person - young person won't engage if parent is spoken to
	Young people have different wants - some are worried about the process, some just see it as a task
	App - note down person's views before transition meetings/appointments
	Identify what stage the young person is at to know what to talk to them about
	If they don't want to engage need to identify additional support they need to engage (e.g. key worker, psychological support)
	Need to have lots of options about how to address this - for some an app might be appropriate, for some you will need to check in on them
	Key worker should be helping the young person prepare
	Include questions that non-verbal people can ask/use with communication device (if they are nervous about asking question)
	For non-verbal people. Use app - to note how they feel beforehand and then asked about what they've written in the app (using AAC)
	Need to include all people in process - need to ask questions directly to them (including non-verbal people)

	Need to have adaptations for people with different impairments (e.g. hearing impairment)
	Training needed for health professionals about how to speak to people at different stages and with different impairments
	It's hard for young people to be involved in the process when there is no process
	Transition key worker should inform you about what to expect
	Outlining the transition process will make you feel less dropped and more engaged
	Create individualized care plan for person transitioning and share with physio
	Planning should start before 16/18
	Needs to be a set age for transition - should be starting post-primary (from when you go to Senior end of school)
PAG	Informal chat between professionals and then young person/family involved in solution rather than part of the process
	Engage young people and families from 16 years in discussions - need to identify the process starts at 16 years
	Transition pathway that service providers and young people/families are all aware of - gives young people/families more power/creates more transparent process
	Every parent should know that 16 years is the age to start the process.
	Logical progression from age 16 years
	Booklet including steps in process for families
	Video about next stage of your life - things to think about e.g., education, - video for service providers and service users
	Video should include person's experience of transition and steps involved
	Video in easy read version outlining things to think about during transition - for service providers and service users
	Service providers to make video to outline their provision during transition - create choice
	Need advance planning for where people are going to go
	Need dedicated age for transition to occur (potentially when domiciliary end) – need definite transition point
Q8	How might we improve knowledge and awareness about CP among healthcare professionals?
YPAG	Health professionals should be mindful of different impairments and severities
	Be aware that severity of CP doesn't indicate the issues that people may experience
	Need to teach health professionals (in undergraduate curriculum) about different disabilities including CP - CP gives a good view of different types of disability

	Asking young people with CP to speak to students - presentation to students about CP by young person
	Need to train GP about CP - GPs often look to person with CP for information - need to start in undergraduate programme
	Older doctors have outdated information
	All health professionals need to know about CP - they may all see people with CP depending on the issues people with CP may have and services they seek
	CP conference (living with CP) hosted by Health Service could create awareness - include young people, adult and older adult talking about CP throughout life
	Need more teaching in physio, OT, medical programmes - invite students in final year to CP conference and speak to people with CP
	HCPs lack education and specialists knowledge of CP
	GPs need more knowledge and education on CP
	GP become like your paediatrician, lucky if you have a good GP but they're not specialized, and may have limited knowledge
PAG	Health professionals do a rotation/placement in CP and adult service
	There should be a adult hospital or service that deals with CP - addresses all issues and knows about CP
	CP clinic for adults
	Annual review with CP clinic
	Education on CP among carers e.g. video, like manual handling
	Education of health professionals
	Video about CP - give general overview
	Video - tips about courtesy when supporting person with CP, providing choices
	Need to respect the dignity for a young person with disability; they are different than supporting an older person with dementia.
	Worry that skills will be lost with configuration of children's services if professionals are seeing people with different conditions. Even if seeing people with CP - there is variation.
	Community adult services may not have expertise even though they try their very best to help and ask what needs are. You miss the expert MDT support that is not available it adulthood.
	Health service need to give staff full time contracts to retain staff who are interested in the area. Benefits families/young people and professionals who build experience
	There is a lack of specialist knowledge, who is responsible for YP, who the right person is
	Health service needs a process to identify who is transitioning, need to pull on data available
Q9	How might we build young people's confidence to make decisions and direct their own care and support over time?

YPAG	Always letting people with CP speak in appointments, from a young age - don't just wait until transition
	Teach people about their CP from young age so they have the language to talk about it and feel confident
	Have peers with CP - match younger person to older person - increase younger person's confidence because they see someone older with it
	Parents might be overprotective - want to protect from societal attitudes/barriers - providing support from peers might make parents less supportive
	Match peers or older/younger people based on interests
	Make young people more aware about living with CP - getting information about living with CP
	Gaining confidence is growing up
	Young people often feel a lack of self-confidence going between school and adult services
	Information needs to be continuous and information needs to be given about what can change as young people get older
	Need more understanding about your body and how to self-manage, need more education
	Checking in and reassessment needed, worrying that people disengage from services because things will change as they age
	Need reassessment of services in adulthood so you're not on your own
	Levels of disability change as you get older and age with CP, you might not get services because of initial level at transfer
	Professionals need to be understanding that it's a process to become confident to speak up
PAG	Talk less to mum and include child from an early age - their viewpoint is important
	At end of conversation with mum, talk directly to the young person
	Start directing conversation/questions to the young person from a young age
	Address emotional wellbeing - will support with confidence
	Talk to young person like they're normal person not unusual
	Tell the young person/use language that they're not unusual
	Acknowledge that the person may have needs but they can be addressed
	Include automatic system where health professionals ask young person if they want to answer questions
	Even if young people are confident, they struggle going into adult services where they're treated completely differently and have no idea what will be expected of them is a stumbling block.
Q10	How might we support young people to develop independence, including independence to direct their care and support throughout the transition process?

YPAG	Parents need to let young people develop independence rather than doing everything for them
	Take small steps to giving young person independence
	Parent gets young person to give their opinions during appointments and steps back
	Treat people with CP like anyone else who is transitioning to adulthood - step back and let them make mistakes/try out things
	Could be parent or health professional to help build independence - but may come from parents more as more opportunities to build independence/health professionals do it as part of their job
	Explaining to young person about their CP is essential
	Other people might need help understanding more about CP and what they can and can't do
	Young people need more education on their diagnosis
	Young people have limited knowledge about CP in general
	Parents may tell you - only when you ask and they don't know about the condition
	Young people need to be made aware of responsibilities in adulthood (e.g. timetable), found taking control hard
	Young people are told they're moving on but are not prepared for what the responsibilities and differences will be
	Need training on responsibilities before you leave school
	An online group for an 1hr for transition might be a way to give people skills
	Need to have groups
PAG	Can do attitude among professionals
	Someone in hospital/service to advocate on behalf of young person so parent doesn't have to
	Young person is given time - not rushing them out - if not enough time, parents jump in to make sure nothing is missed
	Young people given time to make a decision e.g. re medication
	Help parents to step back - health professional tries to support young person to talk (need health professional to be educated and have time)
	Should be allowed to make their own mistakes as they go through the system in order to learn
	Be able to make mistakes
	As they get older, teachers should understand their level (provide more/less support or a push to independence depending on the child's stage)

	Need for personal assistant skill development - how young people interact with people who provide personal care. Need to reframe how those jobs are filled – people may not receive training for the role and often use it as a stop gap job. The role is very intimate, people do it in difficult circumstances for little pay, and their role needs to be valued/appreciated more and given more training
	Need to remove support gradually - growing confidence by removing support
	Not building confidence in childhood/adolescence means having knock backs in adulthood can have a bigger impact
	Allow more independence in adults
	Allowing adults to make choices and deal with consequences,
	Training needs around independence and advocacy in school or groups
	Need peer group
	Developing autonomy and independence will depend on people's ability
	Need to support family and support the young person to have a life outside the family
	Individualized funding may make the transition easier- but it would need to be supported, responsibility can't fall to the family and become a burden for parents
	All adults should have supports to be as independent as possible
	Need to have funding to support aspirations (e.g. be able to provide Assistive technology to support access to employment)
	Need to learn that where they go after school is different
	Have understanding about what adult world is like

d.school, S. (2009). *Design Thinking Bootleg*. In. Retrieved from https://dschool.stanford.edu/resources/design-thinking-bootleg
d.school, S. (2018). Design Thinking Bootleg. https://dschool.stanford.edu/resources/design-thinking-bootleg
Ideo. (2022). *Design Thinking for Educators*. In. Retrieved from https://www.ideo.com/post/design-thinking-for-educators
IDEO.org. (2015). *The Field Guide to Human-Centered Design*. In.
